# Our ways will not change: Future collective continuity increases present prosocial considerations

**DOI:** 10.1111/bjso.12847

**Published:** 2024-12-31

**Authors:** Andrej Simić, Simona Sacchi, Marco Perugini

**Affiliations:** ^1^ Department of Psychology University of Milano‐Bicocca Milan Italy; ^2^ Department of Pedagogy‐Psychology University of Tuzla Tuzla Bosnia and Herzegovina

**Keywords:** future collective continuity, intergroup relations, prosocial behaviour, prosocial beliefs, prosocial intentions

## Abstract

Collective continuity, the perception of the ingroup as an enduring temporal entity, has been linked with ingroup favouritism, negative attitudes and prejudice towards the outgroups. However, previous studies focused mainly on the perceived connection between the past and present of the group. We proposed that the expectation of a strong similarity between the present and future of the national ingroup, *future collective continuity* (FCC), positively affects present intergroup relations construals. In line with the hypotheses, Study 1 (*N* = 202) showed a positive relation between FCC and prosocial outgroup beliefs (i.e., foreigner‐related). Study 2 (*N* = 200) suggested that FCC negatively affects prejudice towards immigrants through lower levels of collective angst. Study 3 (*N* = 250; preregistered) provided experimental evidence that FCC decreased outgroup prejudice and anxiety and increased collective action intentions through collective angst. Furthermore, a moderated mediation model revealed that these effects held only for individuals who identified with their nation more. Our work suggests that believing that the ingroup will not significantly change in the future might make individuals more open towards outgroup members in the present.

## INTRODUCTION

Historically, humans have invested in monuments and art to immortalize their culture and ensure its symbolic continuity (Bennett & Huberman, 2015). This desire to preserve cultural integrity (habits, religion, cuisine) has often fueled aversion towards immigrants across various social and historical settings (Ben‐Nun Bloom et al., 2015; Manevska & Achterberg, 2013). Yet, leaders have also invoked cultural longevity to promote integration. For example, the Roman Emperor Claudius's speech (48 A.D.) argued that admitting Gauls to the Senate would uphold core Roman values, which granted Aedui Gauls senatorial rights (Davis, 1912/2010). Here, we are interested in how these enduring ingroup perceptions shape present outgroup relations.

Individuals experience temporal continuity when they perceive strong links between the present, past, and future (Becker et al., 2018; Block, 1961; Rutt & Löckenhoff, 2016). This coherence provides greater meaning in life (Webster et al., 2021) and buffers against insecurity and stress (Carney & Patrick, 2017; Robbins & Seibel, 2020; Sedikides et al., 2023). Temporal continuity can be perceived on individual and social levels. Self‐continuity benefits well‐being (Kung et al., 2016; Parfit, 1984), while group membership continuity helps confront mortality (Becker, 1973; Pyszczynski et al., 1999), offering a sense of lasting identity even beyond individual existence.

Accordingly, Sani et al. (2007, 2008) define collective continuity as the belief that the group's culture is trans‐generationally transmitted through time (cultural continuity) and that a coherent link between its important historical events exists (historical continuity). Collective continuity helps make the connectivity of the ingroup more real (Sani et al., 2009), fostering group entitativity (Haslam et al., 2000; Sani et al., 2008). Additionally, collective continuity benefits our collective self‐esteem (Sani et al., 2007, 2008, 2009; Smeekes & Verkuyten, 2014a) and well‐being (Jetten & Wohl, 2012; Sani et al., 2009). Therefore, it is no surprise that individuals invest great energy to ensure their groups persist (Sani et al., 2009).

However, that desire to protect the temporal continuity of the ingroup might lead to outgroup selfishness. The literature on past collective continuity (PCC), the perception of the ingroup's past as interconnected, seems to support this hypothesis. For example, perceiving PCC could increase ingroup protection motives (Wohl et al., 2010) and decrease outgroup tolerance (Brewer & Pierce, 2005; Maoulida et al., 2021; Smeekes & Verkuyten, 2014b). Promoting collective nostalgia, an emotion of longing for the ingroup's past (Smeekes et al., 2015; Wildschut et al., 2014), increases positive ingroup evaluations (Cheung et al., 2017; Wildschut et al., 2014) and fosters outgroup prejudice (Smeekes et al., 2015, 2018). Therefore, PCC might act as a buffer to identity threats in times of change (Jetten & Hutchinson, 2011; Obradović & Bowe, 2021; Roth et al., 2017) and make outgroups threatening to positive ingroup values (Smeekes & Verkuyten, 2013).

Perceiving a shared future can also shape perceived collective continuity (Ashmore et al., 2004; Sani et al., 2008). Building on previous work, we aim to study the relationship between collective continuity and intergroup relations in a different direction: the future. Specifically, this work tries to understand how and through which mechanisms projections about the ingroup's future affect intergroup relations.

### The structure of future collective continuity

On the individual level, experiencing future self‐continuity showcases a link between the present and future self (Hershfield, 2011; Parfit, 1984; Sedikides et al., 2023), supports one's well‐being (Adler et al., 2016; Hong et al., 2024) and facilitates positive self‐evaluations (Chu & Lowery, 2024; Sokol & Serper, 2019). Ingroup identification also contributes to self‐esteem (Tajfel & Turner, 1979), suggesting that individuals could connect similarly to the ingroup's future. The extended self‐literature proposes that people integrate objects they identify with into self‐perceptions, with ingroups being among them (Aron et al., 2004; Belk, 1988; Tropp & Wright, 2001). Therefore, the extended self should become a proxy for ingroup identification (Tropp & Wright, 2001), prompting individuals to think about their social and individual selves similarly (Wenzel et al., 2007). Connecting this line of literature to previous theorizing about self (Block, 1961; Sedikides et al., 2023) and collective continuity (Sani et al., 2008, 2009), thinking about self‐ and collective continuity might follow kindred pathways. Thus, we define *future collective continuity* (FCC) as the degree to which one believes that different important events starting from the ingroup's present and ending sometime in the future form a causal and united temporal essence.

Using the *future self‐continuity* (FSC) model (Hershfield, 2011), we provide a possible explanation of how individuals think about the future continuity of their groups. The model posits that a strong connection with the future self can be achieved in at least three ways: by increasing the perceived similarity, vividness, and positive evaluation of the future self. In the same vein, we also understand FCC as a three‐component construct. The similarity with the future collective relates to beliefs that the ingroup will stay similar in the times ahead. We conceptualize the vividness component as the capability to imagine the future of one's ingroup vividly. Finally, the positivity towards the future collective can be understood as the tendency to evaluate the future of the ingroup more positively.

Studies on comparative and future thinking support this hypothesis. People use similarities to compare objects (Corcoran et al., 2011; Posten & Mussweiler, 2017; Todd et al., 2011), and present events influence how individuals shape their ingroup's future (Szpunar & Szpunar, 2016). Envisioning a collective future can also impact present behaviours and judgements, depending on future evaluations (Bain et al., 2013; Judge & Wilson, 2015). Thus, individuals compare the present and future of their ingroup based on expected similarities.

Research on episodic future thinking shows that vivid representations are essential to human cognition (Daniel et al., 2015; Dassen et al., 2016; Gaesser et al., 2019). Vivid imaginations of a shared reality can impact present attitudes and behaviour (Hawlina et al., 2020). One can also assess future events (Newby‐Clark & Ross, 2003). For example, collective emotions like hope are linked to optimistic evaluations of future intergroup relations (Cohen‐Chen et al., 2014; Wohl et al., 2015) and the future in general (Averill et al., 2012; Miceli & Castelfranchi, 2010).

These approaches frame future ingroup thinking as present/future discrepancies, where current values transmit into future contexts (Zittoun & Gillespie, 2018) or future values change the present (Bain et al., 2013; Cohen‐Chen et al., 2014; Wohl et al., 2015). While our approach finds support in these findings, it differs from them because it focuses on transmitting relevant cultural values from the present to the future when examining the effect of collective thinking. Here, we are interested in the degree to which people feel their ingroup will stay similar in the future, can vividly imagine those similarities, and assume a positive outlook towards them.

### FCC and intergroup relations

Considering the individual level, future self‐continuity might be relevant in making people more oriented towards others. Perceiving greater similarities with the future self (Hershfield et al., 2012; Simić et al., 2023) and imagining it vividly (Van Gelder et al., 2013, 2015) could foster moral behaviour. Specifically, orientation towards future self‐schemata might instill greater motivations to maintain a positive present self‐concept (Hershfield et al., 2012; Mazar et al., 2008).

People also show strong inclinations to conserve a positive social self (Cinnirella, 1998; Marques & Paez, 1994; Schmitt et al., 2000; Tajfel & Turner, 1979). Collective continuity can instill pride (Brasil & Cabecinhas, 2017; Denham, 2008; Roth et al., 2017; Sullivan & Dumont, 2014) and reinforce social identity (Van Leeuwen et al., 2013; White & Branscombe, 2019). In the case of PCC, fear of losing positive group continuity might explain defensive behaviours towards ingroups (Brewer & Pierce, 2005; Haraldsson & McLean, 2021; Maoulida et al., 2021). When considering FCC, imagining the future consistency of values and traditions might influence present actions (Glăveanu, 2018) and promote group‐based judgements (Martin et al., 2008; Martin & Gillespie, 2010). FCC may encourage cooperation with other groups, as a collegial future can improve intergroup relations (Badaan et al., 2020; Halperin et al., 2008; Lala et al., 2014) and inspire individuals to reinforce positive ingroup traits in the present (Hopkins et al., 2007; Nunney et al., 2022). Thus, FCC might provide a future‐oriented perspective, potentially alleviating future anxiety for those highly identified with their ingroup.

#### The mediating role of collective angst

Group members experience threats when perceiving dangers to the group's well‐being and values (Stephan & Stephan, 2000). Usually, groups demonstrate negative emotional reactions to threats (Davis & Stephan, 2011; Renfro et al., 2006). A specific type of intergroup threat reaction, collective angst, is composed of fear and anxiety related to the ingroup's future (Wohl & Branscombe, 2009). Perceiving that a present situation could harm the ingroups' future vitality is related to negative attitudes towards outgroups (Jetten & Wohl, 2012; Tabri et al., 2018; Wohl et al., 2010). Furthermore, high levels of collective angst might also motivate ingroup‐strengthening behaviours (Bai & Federico, 2020). On the other hand, perceptions of low ingroup threat are related to lower levels of prejudice (Mancini et al., 2020) and increased intentions to behave prosocially towards the outgroup (Falomir‐Pichastor et al., 2004; Spears et al., 1997).

One possible explanation of why collective angst arises is the fear that the ingroup might lose its distinctive characteristics (i.e., distinction threat, see Roccas & Schwartz, 1993; Wohl et al., 2012). Indeed, globalization (Adams, 2003; Wohl et al., 2010) and ingroup dispersion (Berry, 2003) might trigger collective angst. One possibility to lessen collective angst is highlighting that the group will persevere in the future (Tabri et al., 2018; Wohl et al., 2015). Specifically, FCC might shield groups and individuals against collective angst and thus promote positive ingroup relations.

#### The moderating role of national identity

One's nationhood is an important part of the self‐concept, and it often determines how we shape and conceive our social world (Tajfel & Turner, 1979; Turner & Reynolds, 2001). Accordingly, the Group identity lens model (Major et al., 2003; Verkuyten, 2009) posits that group identity is a reference framework through which individuals process ambiguous social information. Therefore, strong identifiers might interpret being under threat from outside sources. Indeed, a salient national identity might be related to greater threat perceptions (Riek et al., 2006; Van Oudenhoven et al., 1998). Research has shown that high levels of national identification might provoke negative reactions towards outgroup members, such as rejection (Coenders & Scheepers, 2004; Pehrson & Green, 2010), derogation (Inguglia & Musso, 2013; Meeus et al., 2010; Wagner et al., 2012), and xenophobia (Hjerm, 1998; Licata & Klein, 2002). Unlike their low counterparts, high identifiers might react negatively to outgroups they perceive as harmful to their way of life.

However, ingroup love does not always promote outgroup hate (Brewer, 1999; Mummendey et al., 2001). For example, feelings of global fate (Levine & Manning, 2013) and inclusive social identities (Gaertner & Dovidio, 2000; Hornsey & Hogg, 2000) might improve intergroup relations. Another possible strategy for reducing intergroup hostility is alleviating feelings of outgroup threat (Riek et al., 2010; Rios et al., 2018). Therefore, high identifiers might be willing to cooperate with outgroups under certain contexts. One of those conditions would be perceiving FCC. Specifically, FCC could alleviate high identifiers from collective angst by highlighting that the ingroup characteristics they highly value will not change over time.

#### FCC and outgroup collective action

Our theorizing considered how perceptions of FCC might improve intergroup relations. However, such relations could inadvertently reduce motivation for equality and social justice (Wright & Lubensky, 2009). Intergroup contact can sometimes discourage intentions for social change (Cakal et al., 2011; Dixon et al., 2007), potentially damaging disadvantaged outgroups. Disadvantaged members who recognize intergroup inequality may pursue collective action to achieve social change (Van Zomeren, 2016), especially when such actions are fueled by emotions like anger (Van Stekelenburg & Klandermans, 2013) or negative intergroup contact (Brylka et al., 2015; Vala & Pereira, 2020).

Collective action can also come from allies in advantaged groups, driven by solidarity and positive emotions such as sympathy (Becker et al., 2013; Cocco et al., 2024). Ally collective action might help with ingroup negative stereotype management (Hopkins et al., 2007), enhance the ingroup's reputation (Van Leeuwen & Täuber, 2012), and could be common when outgroup suffering threatens the ingroup (Reicher et al., 2006). Perceiving intergroup threats, however, may reduce such actions (Ellemers & Barreto, 2009). These findings suggest that FCC could lessen outgroup threat perceptions and increase collective action for disadvantaged groups, supporting improved intergroup relations.

## THE PRESENT WORK

The current work examines how perceptions of FCC—the belief that an ingroup will maintain its values, beliefs, and traditions over time—affect intergroup relations. FCC posits that despite some inevitable changes (e.g., structural shifts, technological advancements), core ingroup characteristics will persist, potentially fostering positive intergroup behaviour. This approach connects the literature on future self‐continuity and moral behaviour (Hershfield et al., 2012; Simić et al., 2023) with the extended self‐approach (Aron et al., 2004; Tropp & Wright, 2001), collective future thinking (Szpunar & Szpunar, 2016), and imagination literature (Glăveanu, 2018; Zittoun & Gillespie, 2018). We aim to primarily focus on the FCC similarity component and its relationship with intergroup relations construals. Specifically, this FCC component addresses enduring group features (e.g., customs, beliefs, and traditions) and should be connected to how individuals perceive their environment. However, we also measured the other two components (vividness and positivity) to explore the structure of FCC and assess their relationship to intergroup relations in an exploratory fashion.

Our studies explored FCC's effect on intergroup relations construals at different levels (see Alonso‐Arbiol et al., 2023; Burrows et al., 2022). We also explored the underlying mechanisms through which FCC might affect intergroup relations. Namely, in Study 1, we provided preliminary evidence of positive relations between FCC and prosocial beliefs towards the outgroup (Hopkins et al., 2007). Study 2 extended these results to include normative expectations (i.e. prejudicial attitudes; McConahay, 1986) and analysed how collective angst mediates the hypothesized effects. Study 3, using an experimental design, also examined the moderating role of national identity and FCC's effect on outgroup anxiety and collective action intentions (Stephan & Stephan, 1985; Tausch et al., 2011).

While previous work has highlighted the negative effect of PCC on intergroup relations (Malouida et al., 2021; Smeekes & Verkuyten, 2014b; Wohl et al., 2010), we expected that FCC might improve intergroup relations. Our reasoning is rooted in work highlighting the role of collective futures in driving present‐day actions (Bain et al., 2013; Glăveanu, 2018; Judge & Wilson, 2015; Morselli, 2013). Specifically, people with FCC might be motivated to behave cooperatively to ensure a future where the ingroup's characteristics will remain the same (Badaan et al., 2020; Halperin et al., 2008; Lala et al., 2014; Wlodarczyk et al., 2017) and maintain a positive group identity (Hellmann et al., 2015; Hopkins et al., 2007; Nunney et al., 2022; Tung, 2019; Van Leeuwen & Täuber, 2010, 2012; Wakefield & Hopkins, 2017). Our central hypothesis assumes that FCC fosters positive intergroup relations (H1) in the form of its different construals: beliefs (H1a), attitudes (i.e., normative expectations; H1b), and anxiety (H1c). Furthermore, we expect the effect of FCC to go beyond improving intergroup relations and increase behavioural intentions to help the outgroup. By following outgroup collective action (Becker et al., 2013, 2019; Cocco et al., 2024; Hässler et al., 2020; Kende et al., 2021) and outgroup helping research (Charnysh et al., 2015; Reicher et al., 2006; Van Leeuwen, 2007; Van Leeuwen et al., 2013; Van Leeuwen & Täuber, 2012), FCC might highlight that outgroup assistance might help to maintain positive ingroup aspects. Therefore, we expect FCC to increase collective action intentions (H1d). FCC might affect intergroup relations, decreasing the future‐induced ingroup anxiety (Roccas & Schwartz, 1993; Wohl et al., 2012). Because lower levels of collective angst are linked with a more relaxed stance towards outgroup members (Falomir‐Pichastor et al., 2004; Jetten & Wohl, 2012; Spears et al., 1997; Tabri et al., 2018; Wohl et al., 2010), we expect a significant mediational effect of this construct when considering the relationship between FCC and outgroup relations (H2). Finally, while high identifiers are associated with greater perceived intergroup threat (Riek et al., 2006; Van Oudenhoven et al., 1998), threat perceptions can be decreased by highlighting that the ingroup is not in danger (Spiegler et al., 2022; Yogeeswaran & Dasgupta, 2014). Therefore, we expect that the positive effect of FCC on collective angst and intergroup relations should be more relevant for individuals with high national attachment (H3).

The local Ethics Committee approved the studies, and consent was obtained from all participants. We conducted the studies under the Declaration of Helsinki guidelines and described the sampling plan, all data exclusions, and all measures for each study. We analysed data for all three studies in the R statistical software (R Core Team, 2023) using the basic stats, psych (Revelle, 2019), GPArotation (Bernaards & Jennrich, 2005), lavaan (Rosseel, 2012), effectsize (Ben‐Shachar et al., 2020) and interactions (Long, 2019) packages. Datasets, analysis code, and research materials are available at https://osf.io/ezy5p/?view_only=efaff11e2fc347a5877d6d07edb7773a.

## STUDY 1

Study 1 aims to explore the correlational relationships between the components of FCC and prosocial behaviour beliefs towards the outgroup. Additionally, our goal was to provide evidence of the hypothesized structure of the FCC.

### Participants

All Italian participants were eligible for participation. We invited participants via an online survey link shared on social media platforms. A total of 223 participants took part in the study. When we eliminated careless responses (see below), our sample consisted of 202 valid participants (*N*_male_ = 71, *N*_female_ = 128, *N*_other_ = 3, *M*_age_ = 38.06, *SD*_age_ = 13.88). We have conducted a sensitivity analysis using Monte Carlo simulations for indirect effects (Schoemann et al., 2017). When power is set at 0.80 and *α* = .05, with the mentioned sample size, it is possible to detect a significant indirect effect (.05) for a simple mediation model (Model 4; Hayes, 2018) even when the correlations between the variables are relatively low (*r* = .26).

### Materials and procedure

The study was administered on the online platform Qualtrics. After providing informed consent, participants answered questions about their demographic information. They filled the following measures: the Future collective continuity questionnaire (FCCQ), the Prosocial Beliefs Towards the Outgroup Scale ([PBOS]; Hopkins et al., 2007), and a measure to detect careless responses in long surveys ([SRSI]; Meade & Craig, 2012). A description of the measures used in Study 1 follows.

#### Future collective continuity questionnaire (FCCQ)

We adapted the Future Self‐Continuity Questionnaire (Sokol & Serper, 2020) to measure FCC facets. For our purposes, the scale was adapted to measure the proposed components of FCC by replacing the target word “you” with “Italians.” Furthermore, in each item, we replaced “10 years from now” with “50 years from now” because most individuals consider that societal change is plausible in 50 years (Bain et al., 2013). The adapted measure is composed of three subscales: future collective similarity (4 items; How similar are Italians now to what they will be like 50 years from now?), future collective vividness (3 items; How vividly can you imagine what Italians will be like in 50 years from now?), and future collective positivity (3 items; Do you like what Italians will be like 50 years from now?). Participants provided their answers using a seven‐point scale (1 = completely different/not at all, 7 = exactly the same/completely).

#### Prosocial Outgroup Beliefs Scale (PBOS, Hopkins et al., 2007)

We used a scale developed initially by Hopkins et al. (2007) to measure participants' beliefs about the prosocial behaviours of their ingroup towards outgroup members (i.e., foreigners). In this three‐item measure, we asked participants to think about a typical ingroup member (Italian) and respond how much the behaviours listed in the items (e.g., Helping a fellow foreigner in distress) are representative of the Italian group on a 7‐point scale (1 = strongly disagree, 7 = strongly agree).

#### Careless responses measure

We used the Self‐Reported Single Item (SRSI; Meade & Craig, 2012) to detect and filter out participants who responded carelessly. At the end of the survey, participants responded to a yes/no question about whether they think their data should be used in further analyses. We excluded participants who answered with “no.”

### Results

As can be seen from Table 1, all variables were measured with scales showing good to excellent internal consistencies. To test our proposed three‐factor structure, we ran a confirmatory factor analysis (CFA) using the maximum likelihood estimation method and evaluated the model based on the recommendations by Hu and Bentler (1999) and Hair et al. (2019). The three‐factor model showed acceptable fit, CFI = 0.98, RMSEA = 0.08, SRMR = 0.04. Additionally, we examined the internal construct validity of our model. All individual standardized item loadings exceeded the value of 0.70 (Hair et al., 2019). All factors showed acceptable composite reliability (CR_similarity_ = 0.78, CR_vividness_ = 0.73, CR_positivity_ = 0.73) and average variance extracted (AVE_similarity_ = 0.82, AVE_vividness_ = 0.81, AVE_positivity_ = 0.79) indices suggesting good internal convergent validity. We also found evidence of good internal discriminant validity. The maximum shared variances for each factor (MSV_similarity_ = 0.34, MSV_vividness_ = 0.34, MSV_positivity_ = 0.29) were smaller than the corresponding AVEs. As theoretically expected, the similarity factor correlated moderately and positively with both vividness (*r* = .59, *p* < .001) and positivity (*r* = .37, *p* < .001). Likewise, vividness positively correlated with the positivity factor, *r* = .54, *p* < .001. We also tested a global, one‐factor model, which showed an unacceptable fit, CFI = 0.59, RMSEA = 0.33, SRMR = 0.20, and fitted the data much worse than the three‐factor model, *χ* ^2^(3) = 734.52, *p* < .001. Overall, the three‐factor model showed an acceptable model fit and construct validity and was clearly superior to a single general‐factor model.

**TABLE 1:** Study 1: Means, standard deviations, reliability coefficients, and correlations of the study variables.

Variable	*M*	*SD*	*α*	1	2	3	4
Similarity (1)	3.89	1.17	.94				
Vividness (2)	3.66	1.01	.94	.57**			
Positivity (3)	3.90	0.99	.92	.35**	.49**		
Prosocial beliefs towards the outgroup (4)	4.27	1.26	.91	.31**	.21**	.40**	

*Note*: *M* and *SD* represent the means and standard deviations. *α* represents the internal consistency coefficient for each scale.

*p < .05; **p < .01.

As Table 1 suggests, all three components were positively related to outgroup prosocial behaviour beliefs. The correlations between similarity and vividness with outgroup prosocial beliefs were relatively small, while positivity achieved a moderate correlation with the same variable. Additionally, a linear multiple regression model containing the three FCC components significantly predicted outgroup prosocial beliefs, *R* ^2^ = .23, $R_{\mathrm{adj}}^{2}$ = .22, *F*(3, 198) = 20.11, *p* < .001. When evaluating individual predictor contribution, we identified a significant effect of the similarity, *b* = .37, *t*(198) = 4.32, *p* < .001, $r_{\mathrm{sp}}^{2}$ = .07; and positivity components, *b* = .36, *t*(198) = 3.79, *p* < .001, $r_{\mathrm{sp}}^{2}$ = .06. However, vividness did not predict outgroup prosocial beliefs, *b* = −.02, *t*(198) = −0.21, *p* = .832, $r_{\mathrm{sp}}^{2}$ < .01. In other words, Study 1 provides preliminary evidence that FCC might foster beliefs about outgroup prosociality.

## STUDY 2

Our approach in measuring outgroup prosociality in Study 1 was to assess participants' beliefs of how a typical ingroup member behaves. These deliberations might result from greater ingroup idealization (Golec de Zavala et al., 2009; Leach et al., 2007; Roccas et al., 2006) and could thus reflect an unrealistic view of the ingroup. Study 2 addresses this concern by assessing individuals' expectations of whether a typical ingroup member *should* behave positively to the outgroup. Specifically, we aim to study how FCC components relate to prejudice towards outgroup members. While in Study 1, we focused on an ambiguous outgroup (i.e., foreigners), in Study 2, we decided to measure prejudice towards a more threatening outgroup, immigrants living in Italy. Specifically, recent work highlighted that Italians perceive immigrants as a salient outgroup (Dixon et al., 2018; European Commission, 2019). This outgroup might instill a threat related to Italian values and norms (Salvati et al., 2020), further amplified by media (Vaes et al., 2015) and politics (Ambrosini, 2013). On the other hand, one could also define immigrants living in Italy as a disadvantaged group since immigrant families represent almost a third of families living in poverty in Italy (Italian Caritas and the Migrantes Foundation, 2023).

### Method

#### Participants

All participants who identified as Italian were eligible to participate in the study. A total of 271 participants accessed the online study. When removing careless responses, our sample consisted of 200 participants (*N*_male_ = 77, *N*_female_ = 121, *N*_other_ = 2, *M*_age_ = 36.95; *SD*_age_ = 12.88). By following Schoemann et al. (2017), a sensitivity power analysis showed that it is possible to detect a significant indirect effect (.05) for a simple mediation model (Model 4; Hayes, 2018) when considering low correlations between the variables (*r* = .26) while setting power at 0.80 and *α* at .05.

#### Materials and procedure

The study was administered on an online platform (Qualtrics) to share and conduct surveys. After giving consent, participants provided their demographic information and completed the following measures: FCCQ, the adapted Modern Prejudice Scale ([MPS]; McConahay, 1986), the Collective Angst Scale ([CSA]; Wohl & Branscombe, 2009), the SRSI, and other measures outside the scope of this work. The study lasted approximately 10 min.^1^ A brief description of the measures not used previously follows.

##### Modern Prejudice Scale ([MPS]; McConahay, 1986)

The MPS was developed to measure individual differences in covert racial attitudes of White Americans towards African Americans. We adapted the items to measure expectations of the ingroup behaviour towards immigrants living in Italy. Participants answered nine items (e.g., Italians *should* mix with immigrants) using a 7‐point scale (1 = never, 7 = always).

##### Collective Angst Scale ([CAS]; Wohl & Branscombe, 2009)

CAS measures individual differences in concerns that the ingroup's existence is and will be threatened in the future. Participants responded on a 7‐point scale (1 = completely disagree, 7 = completely agree) on a set of five items describing concerns about the safety of the ingroup and its members' way of life (e.g., I am concerned about external threats to the Italian way of life).

### Results

Table 2 shows the internal consistencies of all measures ranging from acceptable to excellent. All three FCC components were negatively related to modern prejudice towards immigrants and collective angst. As expected, negative attitudes towards the outgroup were positively related to collective angst.

**TABLE 2:** Study 2: Means, standard deviations, reliability coefficients, and correlations of the study variables.

Variable	*M*	*SD*	*α*	1	2	3	4	5
Similarity (1)	3.71	1.27	.86					
Vividness (2)	3.52	1.08	.90	.45**				
Positivity (3)	3.74	1.30	.93	.38**	.50**			
Outgroup prejudice (4)	2.12	0.93	.78	−.29**	−.35**	−.38**		
Collective angst (5)	2.12	1.24	.89	−.32**	−.32**	−.36**	−.14*	

*Note*: *M* and *SD* represent the means and standard deviations. *α* represents the internal consistency coefficient for each scale.

*p < .05; **p < .01.

**FIGURE 1:**
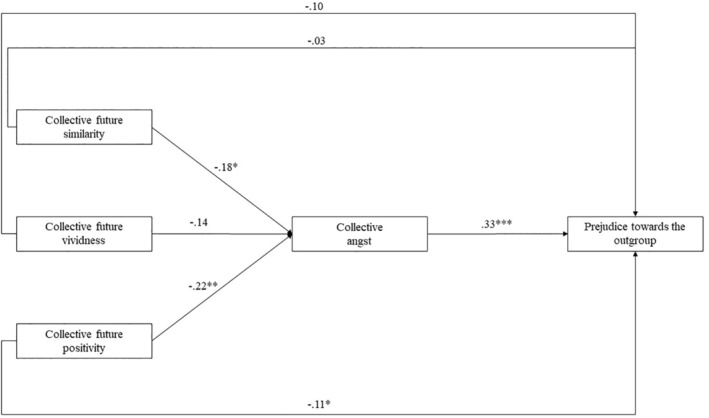
Study 2: Mediation model containing FCC components are predictors, outgroup prejudice and ingroup prosocial behaviour as outcome variables, and collective angst as a mediator. The figure contains unstandardized regression weights as representations of path coefficients. *p < .05; **p < .01; ***p < .001.

We tested a mediation model containing FCC components as predictors of modern prejudice while collective angst served as a mediator. As Figure 1 shows, similarity and positivity were significant predictors of collective angst. Furthermore, collective angst significantly predicted modern prejudice. When estimating the significance of the indirect paths using the bootstrapping method (10,000 bootstrapped samples), a significant mediation effect of collective angst emerged when considering the similarity, *b* = −.06, *β* = −.08, 95% CI [−.11, −.01], and positivity components, *b* = −.07, *β* = −.05, 95% CI [−.14, −.02]. However, collective angst did not mediate the effect of FCC vividness, *b* = −.05, *β* = −.10, 95% CI [−.12, .02]. The results suggest that FCC might be positively linked with normative outgroup prosocial expectations. Furthermore, lower levels of collective angst seemed to mediate this effect in the case of FCC similarity and positivity.

## STUDY 3

While Study 2 provided further evidence that FCC might dampen negative outgroup attitudes, it still lacked experimental data to corroborate our hypotheses. In Study 3 we manipulated FCC similarity to examine its causal effect on outgroup attitudes and replicate the mediation effect of collective angst. We also explored how national identity moderates our experimental effect. Finally, we aimed to go beyond measures of normative expectations and extend our work to anxiety and fear‐related emotions (Barbalet, 1996; Forgas, 2001; Stephan & Stephan, 1985). We preregistered Study 3 via AsPredicted (https://aspredicted.org/94X_PY7), reporting the study design, the planned sample size, the inclusion/exclusion criteria, and planned analyses.

### Method

#### Participants

All participants over 18 who identified themselves as Italian were eligible for participation. As preregistered, we aimed to recruit at least 250 participants that would allow us to detect an effect size of *f* ^2^ = 0.032 in a model with three parameters (a continuous predictor, a binary predictor, and their interaction) when setting power at 0.80 and α = .05. A total of 254 participants took part in the study. After removing careless responses using the SRSI, we were left with 252 responses (*N*_male_ = 91, *N*_female_ = 159, *N*_other_ = 2, *M*_age_ = 28.87, *SD*_age_ = 12.44).

#### Materials and procedure

We created and administered the online survey on Qualtrics. After giving consent, we collected demographic data and asked participants to fill out the National Identification Scale ([NIS]; Roccas et al., 2006) and a seven‐point scale item measuring political orientation (higher scores reflect a stronger right‐winged orientation). Next, we randomly assigned participants to a continuity (*N* = 126) or a discontinuity (*N* = 126) condition. Following the general understanding of collective cultural continuity as the perseverance of ingroup aspects (Sani et al., 2007), we attempted to manipulate FCC by emphasizing that important (Italian) cultural symbols will endure/wane with time. Specifically, both groups watched three timelapse videos in randomized order showcasing three different symbols of Italian culture (the Italian flag, Piazza Venezia in Rome, and the tower of Pisa) as they moved through time 50 years into the future (from 2023 to 2073). The symbols and the surrounding environment remained the same in the continuity condition, highlighting cultural continuity. The videos in the discontinuity condition changed through time (e.g., futuristic buildings, transport measures, and the disappearance of classical architecture).^2^ Then, participants needed to spend at least 2 min on a letter‐writing task where they listed five ways Italians will stay the same (continuity condition) or different (discontinuity condition) 50 years in the future. After completing their respective tasks, all participants filled out the FCCQ, MPS, CAS, outgroup anxiety scale ([OAS]; Tausch et al., 2007; Voci, 2006), collective action intentions scale ([CAIS]; Lantos et al., 2020; Tausch et al., 2011), and the SRSI. The study lasted approximately 15 min. A brief description of the measures not used previously follows.

##### National Identification Scale ([NIS]; Roccas et al., 2006)

The scale is composed of two subscales: ingroup attachment (8 items, e.g., “I love Italy”) and ingroup glorification (8 items, e.g. “The Italian army is the best army in the world”). In this study, we merged the two subscales to form a composite of ingroup attachment. Participants needed to answer each item on a 7‐point Likert scale (1 = strongly disagree, 7 = strongly agree), where higher scores indicate a greater feeling of attachment and a tendency to glorify the national group.

##### Outgroup anxiety scale (OES)

We combined items from the Intergroup anxiety scale (Tausch et al., 2007) and Outgroup trust scale (Voci, 2006) to measure anxiety and fear‐related emotions. Participants were asked to think about the outgroup members and rate how they experienced them on a set of five negative (afraid, anxious, nervous, worried, suspect) and two positive (trust, positive expectations) emotions using a seven‐point scale (1 = not at all, 7 = extremely). Higher scores reflected greater anxiety towards outgroup members.

##### Collective action intentions scale (CAIS)

We measured outgroup collective action intentions by following previous work measuring the same construct in similar contexts (Lantos et al., 2020; Tausch et al., 2011). Participants needed to read six items (e.g., attending lectures about improving the immigrants' position in Italy) and answer following a seven‐point scale (1 = not likely at all, 7 = very likely).

### Results

#### Correlational analysis

First, we aimed to replicate the correlational patterns from Study 2. As seen in Table 3, all measures showed acceptable to excellent internal consistency. FCC Similarity correlated negatively with modern prejudice, collective angst, and outgroup anxiety and positively with collective action intentions and national attachment. FCC Vividness correlated positively with modern prejudice, collective angst, national attachment and political orientation. Finally, we observed a negative correlation between the positivity component and collective angst. The same variable correlated positively with national attachment and political orientation.

**TABLE 3:** Study 3: Means, standard deviations, reliability coefficients, and correlations of the study variables.

Variable	*M*	*SD*	*α*	1	2	3	4	5	6	7	8	9
Similarity (1)	3.99	1.24	.84									
Vividness (2)	3.72	1.36	.93	.29**								
Positivity (3)	3.79	1.38	.94	.22**	.45**							
Outgroup prejudice (4)	2.40	1.15	.73	−.28**	.19**	−.03						
Collective angst (5)	2.71	1.48	.86	−.23**	.12*	−.14*	.58**					
Outgroup anxiety (6)	3.22	1.28	.79	−.28**	.11	−.08	.70**	.61**				
Collective action intentions (7)	3.55	1.68	.86	.14*	−.06	.07	−.65**	−.48**	−.62**			
National identity (8)	3.98	1.33	.76	.14*	.45**	.42**	.42**	.35**	.36**	−.38**		
Political orientation (9)	3.75	1.58		.00	.31**	.16**	.61**	.43**	.48**	−.60**	.63**	

*Note*: *M* and *SD* represent the means and standard deviations. *α* represents the internal consistency coefficient for each scale. We could not calculate *α* for the variable “Political orientation” since it represents a one‐item measure.

*p < .05; **p < .01.

#### Manipulation check

Table 4 contains descriptive statistics of our main study variables based on condition. A one‐way MANOVA suggested significant group differences on a linear combination of the three FCC components, *F*(3, 248) = 17.84, *V* = 0.18, *p* < .001, *η* ^2^ = .18. We found that participants in the continuity condition scored higher in FCC Similarity than their discontinuity counterparts, *t*(250) = 7.13, *p* < .001, *d* = 0.90. Participants did not differ in vividness, *t*(250) = 0.94, *p* = .347, *d* = 0.12; and positivity scores, *t*(250) = 0.06, *p* = .952, *d* = 0.01. The results suggest that we successfully affected the similarity but not the other two FCC components.

**TABLE 4:** Study 3: Means and standard deviations of the main study variables for the continuity (*N* = 126) and discontinuity (*N* = 126) condition.

Variable	Continuity condition		Discontinuity condition	
	*M*	*SD*	*M*	*SD*
Similarity	4.50	1.13	3.48	1.14
Vividness	3.80	1.38	3.63	1.33
Positivity	3.80	1.50	3.79	1.26
Outgroup prejudice	2.29	0.95	2.52	1.32
Collective angst	2.43	1.35	2.99	1.55
Outgroup anxiety	2.93	1.04	3.51	1.43
Collective action intentions	3.72	1.52	3.38	1.81

#### Direct manipulation effects

As preregistered, we performed a series of four independent sample *t*‐tests to test our manipulation's effect on collective angst, outgroup prejudice, outgroup anxiety, and collective actions and intentions. As shown in Table 4, we found that the continuity condition reported lower levels of collective angst, *t*(250) = −3.04, *p* = .002, *d* = −0.38; and outgroup anxiety, *t*(250) = −3.65, *p* < .001, *d* = −0.46. However, we found no significant manipulation effects on modern prejudice, *t*(250) = −1.64, *p* = .103, *d* = −0.21; and collective action intentions, *t*(250) = 1.59, *p* = .113, *d* = 0.20, although in both cases the direction of the differences was in line with expectations.

Going further from the preregistration, we were interested in whether our effects persisted or changed when the analysis included participants' political orientation as a covariate. The effects on collective angst, *F*(1, 248) = 11.67, *p* < .001, $\eta_{p}^{2}$ = .04; and outgroup anxiety, *F*(1, 248) = 17.38, *p* < .001, $\eta_{p}^{2}$ = .07; persisted when controlling for political orientation. Additionally, after including political orientation, the manipulation significantly affected both modern prejudice, *F*(1, 248) = 4.18, *p* = .042, $\eta_{p}^{2}$ = .02; and collective action intentions, *F*(1, 248) = 4.24, *p* = .040, $\eta_{p}^{2}$ = .02.

#### Serial mediation model

**FIGURE 2:**
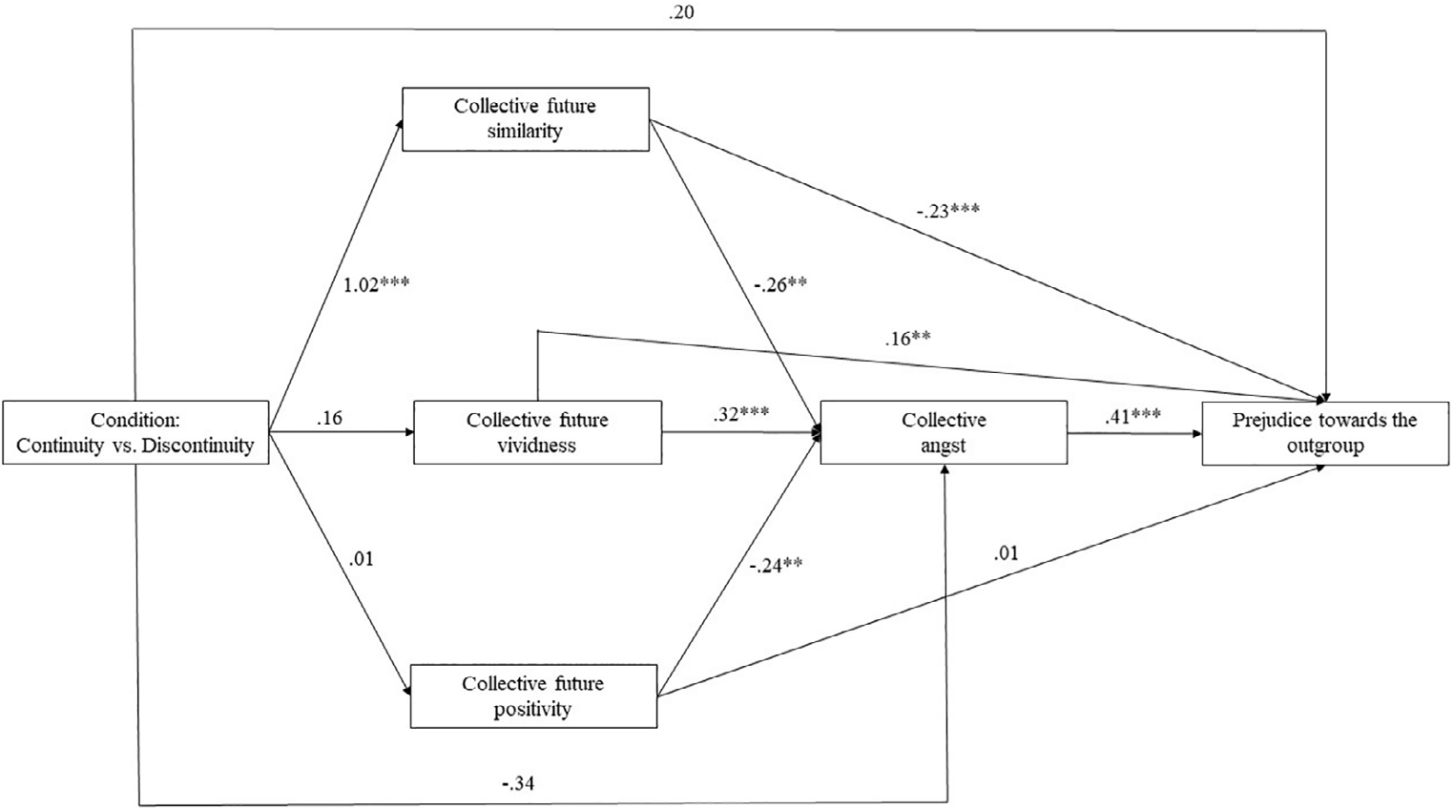
Study 3: Mediation model containing Condition as the predictor, FCC components as the first mediators, collective angst as the second mediator, and outgroup prejudice as the outcome variable. The figure contains unstandardized regression weights as representations of path coefficients. *p < .05; **p < .01; ***p < .001.

We fitted a serial mediation model with condition as the independent, FCC components as three parallel first mediators, collective angst as the second mediator, and outgroup prejudice as the dependent variable (see Figure 2). Replicating the patterns identified in Study 2, similarity was a significant predictor of collective angst, which predicted modern prejudice. Additionally, condition (continuity vs. discontinuity) significantly predicted the similarity component. Finally, FCC vividness positively affected collective angst and modern prejudice. Table 5 contains the tested indirect paths following the bootstrapping method (10,000 bootstrapped samples). Only two indirect paths emerged as significant: the path through collective future similarity and through both the similarity component and collective angst. In other words, the mediation model indicates that higher levels of collective future similarity and lower collective angst might explain the negative effect of condition on outgroup prejudice.^3^

**TABLE 5:** Study 3: Estimated indirect paths of the mediation model containing Condition as the predictor, CFC components as the first mediators, collective angst as the second mediator, and outgroup prejudice as the outcome variable.

Indirect effect	*b*	LCI	UCI
**Condition → collective future similarity → outgroup prejudice**	**−.24**	**−.41**	**−.12**
Condition → collective future vividness → outgroup prejudice	.03	−.02	.10
Condition → collective future positivity → outgroup prejudice	.00	−.02	.02
Condition → collective angst → outgroup prejudice	−.14	−.31	.02
**Condition → collective future similarity → collective angst → outgroup prejudice**	**−.11**	**−.21**	**−.04**
Condition → collective future vividness → collective angst → outgroup prejudice	.02	−.02	.08
Condition → collective future positivity → collective angst → outgroup prejudice	.00	−.04	.04

*Note*: *b* = value of the unstandardized regression coefficient. 95% LCI = lower bound of the 95% confidence interval for the estimated indirect effects. 95% UCI = upper bound of 95% confidence interval for the estimated indirect effects. Confidence intervals that do not contain zero are indicative of a significant indirect effect. The significant indirect effects are bolded.

#### Moderated mediation models

**FIGURE 3:**
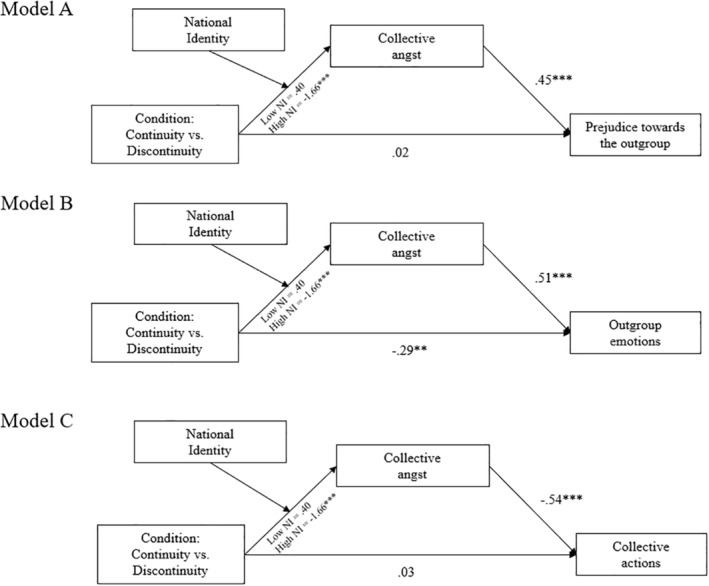
Study 3: Moderated mediation models containing Condition as the predictor, collective angst as the mediator, and outgroup prejudice (Model 1), Outgroup emotions (Model 2), Collective actions (Model 3) as the outcome variables. The figure contains unstandardized regression weights as representations of path coefficients. *p < .05; **p < .01; ***p < .001.

We fitted three moderated mediation models (Model 7; Hayes, 2018) containing manipulation as the independent, national attachment as a moderator, collective angst as a mediator, and outgroup prejudice (Model 1), outgroup anxiety (Model 2), collective actions intentions (Model 3) as dependent variables (see Figure 3).^4^ Before running the analysis, we mean‐centred scores on the NIS (Baron & Kenny, 1986). Table 6 shows that the overall mediation effect for all three models was significant, indicating that lower levels of collective angst mediate our manipulation effect on the dependent variables. Furthermore, we found support for all three moderated mediation models with confidence intervals of the moderated mediation indices not containing zero. When inspecting this effect further, we identified that the mediation effect of collective angst was significant for higher, but not lower identifiers. In line with our hypotheses, the results suggest that our manipulation might affect outgroup relations construals when considering high identifiers through collective angst (Table 6).

**TABLE 6:** Study 3: Estimated conditional indirect effects of collective angst when considering Condition as the predictors and outgroup prejudice (Model 1), outgroup emotions (Model 2), and collective actions (Model 3) as outcome variables.

Indirect effect	Outgroup prejudice (model 1)			Outgroup emotions (model 2)			Collective actions (model 3)		
	*b*	LCI	HCI	*b*	LCI	HCI	*b*	LCI	HCI
Index of moderated mediation	−.36	−.48	−.25	−.40	−.56	−.28	.43	.29	.59
Estimated indirect effect									
Overall	−.29	−.46	−.14	−.31	−.51	−.16	.34	.17	.56
Lower levels of national identity (−1 *SD*)	.18	−.02	.41	.21	−.03	.47	−.22	−.51	.02
Higher levels of national identity (+1 *SD*)	−.75	−1.00	−.54	−.84	−1.12	−.61	.90	.64	1.20

*Note*: *b* = value of the unstandardized regression coefficient. LCI = lower bound of the 95% confidence interval for the estimated indirect effects. UCI = upper bound of 95% confidence interval for the estimated indirect effects. Confidence intervals that do not contain zero are indicative of a significant indirect effect.

## DISCUSSION

Building on the literature on the extended self (Aron et al., 2004; Belk, 1988; Clayton et al., 2015; Tian & Belk, 2005), we propose that maintaining a positive social identity may be just as beneficial as sustaining a consistent positive personal identity (Tropp & Wright, 2001; Wenzel et al., 2007). By reconciling this line of work with the FSC model (Hershfield, 2011), the same pathways relevant to understanding future self‐continuity might also be applicable when considering future continuity on the collective level. We labelled this construct FCC and studied how it affects present intergroup relations.

### FCC and intergroup relations

Studies on PCC suggest that perceiving strong temporal links within an ingroup's past can increase the desire to protect it (Brewer & Pierce, 2005; Smeekes & Verkuyten, 2014a) and reduce tolerance for outgroups (Malouida et al., 2017; Roth et al., 2017; Wohl et al., 2010). Our studies examined how FCC relates to four intergroup constructs. In Study 1, all three FCC components positively correlated with outgroup prosocial beliefs (H1A), meaning that FCC similarity, vividness, and positivity predicted stronger expectations of prosocial behaviour towards outgroups. Study 2 extended these findings, showing that FCC facets negatively correlated with normative prosocial expectations towards the outgroup (H1B), indicating that expecting a similar, vivid, and positive ingroup future might be related to perceptions that a typical ingroup member *should* behave prosocially towards the outgroup. Finally, Study 2 provided correlational evidence of our mediation hypothesis (H2): lower levels of collective angst explained the effect of FCC similarity.

Study 3 explored FCC's effects on outgroup anxiety (H1C) and collective action intentions (H1D). Our FCC manipulation decreased collective angst and outgroup anxiety and, when controlling for political orientation, affected outgroup prejudice and collective action intentions. A serial mediation model demonstrated that FCC manipulation achieved its effect through greater levels of FCC similarity and lower collective angst (H2). Although we appreciate that the first mediator was also a manipulation check measure, a recommended practice in mediation analysis is to show that a manipulation check and the dependent variables correlate (Lench et al., 2014; see also Ejelöv & Luke, 2020). This method allows for testing whether differences in FCC caused by our manipulation can predict reported collective angst and outgroup prejudice (Lench et al., 2014).^5^

Our moderated mediation models indicated that this effect held primarily for high national identifiers (H3), with collective angst playing a key mediating role. Specifically, our manipulation seems to achieve its hypothesized effects only when considering high identifiers. In the case of participants with lower levels of national identity, the estimated mediation model was not significant. Additionally, our exploratory analysis identified political identity as another moderator of our experimental effect. Because national identity and political orientation correlated in Study 3 and previous work (see Osborne et al., 2017; Roccas et al., 2010; Roccas & Sagiv, 2010), significant condition effects were in the same direction and with a similar magnitude when considering right‐wing oriented participants (see Supplemental materials). Therefore, Study 3 provided experimental evidence that FCC might affect different intergroup relations, emotions, and outgroup collective action.

Our three studies suggest the similarity component was consistently linked with outgroup relations construals. Results indicate that FCC similarity is relevant for fostering openness to outgroups, consistent with findings on the self‐continuity level (Hershfield et al., 2012; Simić et al., 2023). In other words, while individuals can imagine the ingroups' future vividly (Bain et al., 2013; Shrikanth et al., 2018; Yamashiro & Roediger, 2019), to become more open to the other groups, they might first need to believe in the ingroups' essential characteristics temporal stability.

### What is different between FCC and PCC?

FCC could affect intergroup relations differently than PCC. While both constructs highlight the ingroup as a lasting entity over time, we hypothesized that FCC might lower the feelings of collective angst, especially in those who identify with their nation more.

Believing in a stable ingroup future may make people more open to outgroups (Bain et al., 2013; Glăveanu, 2018; Judge & Wilson, 2015; Morselli, 2013). This openness could help preserve valued ingroup traits (Cinnirella, 1998; Tajfel & Turner, 1979) and maintain (positive) social identities (Hopkins et al., 2007). By being open, people show that their group is warm (Van Leeuwen & Täuber, 2012), competent (Hellmann et al., 2015), and enhance its reputation (Wakefield & Hopkins, 2017). Reduced collective angst, facilitated by FCC, may further predict positive outgroup attitudes (Falomir‐Pichastor et al., 2004; Wohl et al., 2012). FCC could thus ease anxieties about the ingroup's future.

Our work suggests that the effect of FCC goes beyond intergroup relations and extends to, at least, intentions to provide outgroup help. By showing that FCC increased outgroup collective actions, the studies corroborate the literature on allyship and collective action (Becker, 2012). It is also important to note that we measured outgroup collective action towards an underprivileged population in Italy: immigrants. Because intergroup threat decreases allyship intentions (Ellemers & Barreto, 2009; Shepherd et al., 2018), FCC might help people see (Cocco et al., 2024; Hässler et al., 2020) and identify with (Becker et al., 2013, 2019; Kende et al., 2021) the problems of the underprivileged more by lowering collective angst.

Our findings primarily apply to high national identifiers, as our moderated mediation models suggest. Low national identifiers typically show less ingroup defensiveness (Stevenson & Manning, 2010; Ziemes et al., 2019) and are more open to outgroups (Alvarez et al., 2018; Meeus et al., 2010; Verkuyten, 2009). In contrast, those with high national identity tend to be more ingroup‐focused (Duckitt, 2006; Hodson & Busseri, 2012; Riek et al., 2006). Additionally, right‐wing individuals with high national identity may be more past‐focused, which limits their future orientation (Duckitt et al., 2010; Lammers & Baldwin, 2020). Thus, FCC may not influence those already inclined to be prosocial towards outgroups but could help high identifiers become more open to others (Spiegler et al., 2022; Yogeeswaran & Dasgupta, 2014), effectively buffering the negative impact of national attachment on intergroup relations.

The main difference between FCC and collective continuity with a trajectory to the past (PCC) is how each modulates intergroup threat. However, following recent practices in interpreting mediation data (Danner et al., 2015; Lemmer & Gollwitzer, 2017), we are not willing to devalue the effects of other variables that might mediate the relationship between FCC and intergroup construals. The decreased outgroup threat (collective angst) hypothesis might be one of many explaining our identified effect. For example, the FCC could increase the salience of social safety and well‐being needs and thus facilitate positive intergroup relations (Fiske & Rai, 2014; Rai & Fiske, 2011). However, previous studies focusing on PCC found that activating those needs might elevate ingroup protection motivations (Brewer & Pierce, 2005; Smeekes & Verkuyten, 2014a). Social identity theory posits that a way to protect social self‐esteem might be to engage in outgroup discrimination (e.g. Lemyre & Smith, 1985). In that regard, perceptions of PCC might negatively affect outgroup perceptions by highlighting that all that the group *was* is under threat. As previously explained, FCC might foster ingroup relations by emphasizing that outside threats will not destroy everything the group *will be*. One might consider prosocial behaviour as a strategic way to maintain a positive social identity (Cinnirella, 1998; Marques & Paez, 1994; Schmitt et al., 2000; Tajfel & Turner, 1986) in the future. FCC could offer optimism to intergroup relations compared to the fatalistic outlook of PCC. Specifically, maintaining positive intergroup relations might be related to a motivation to sustain and develop the ingroups' integrity and status (Cialdini & Richardson, 1980). Therefore, group members can see prosocial behaviour as a pathway to protect the ingroup (e.g., its future reputation and status).

### Implications and limitations of current research

We expanded the present literature in four main ways. First, rather than focusing on PCC as in previous studies (Brewer & Pierce, 2005; Maoulida et al., 2021; Wohl et al., 2010), we explored FCC. Second, we showed that people can think about a continuous future of their ingroup instead of focusing on present/future discrepancies (Bain et al., 2013; Judge & Wilson, 2015; Shrikanth et al., 2018; Yamashiro & Roediger, 2019). Therefore, the current work presents a novel attempt to connect FCC to intergroup relations. By projecting ingroup continuity, one can foster prosocial tendencies, helping to increase cooperation and trust between different groups. If people believe their ingroup's ways will remain unaffected, it may reduce tensions associated with concerns about its future vitality. This approach is especially relevant for high national identifiers and right‐wing individuals, who are often more sensitive to perceived threats and show more negative intergroup attitudes (Duckitt, 2006; Hodson & Busseri, 2012; Riek et al., 2006; Van Oudenhoven et al., 1998). For these individuals, perceiving a sense of continuity in the ingroup might encourage openness and cooperation with other groups.

Practically, FCC might help manage public discourse and policy. While conservative and high‐identity citizens may resist social progress and inclusion policies (Butz, 2009; Kosterman & Feshbach, 1989; Van Lange et al., 2012), highlighting the continuity of ingroup values could facilitate a more cooperative stance. These findings align with recent work suggesting that emphasizing cultural continuity may be an effective strategy to engage individuals who may otherwise be resistant to social change (Syfers et al., 2021, 2024).

The present work is not without limitations. First, the three study samples consisted solely of Italian participants living in Italy. Because national identity is a context‐specific variable (e.g. Andreouli & Howarth, 2013; Vargas‐Salfate et al., 2020), one should approach with caution when extending these results to other countries and cultures. Second, throughout our studies, we identified relatively modest (small to medium) effect sizes. Therefore, interpreting the strength of possible FCC interventions on intergroup relations should be cautiously approached. However, intergroup attitudes and emotions might resist change (Murrar & Brauer, 2019), and one possibility to instill intergroup behaviour change might be to affect social norms and policies (Brauer, 2024; Murrar et al., 2020). Focusing on social policies highlighting FCC might help accumulate these small effects over time (Ellis, 2010). Third, our work focused on beliefs, expectations and specific intentions that do not always successfully translate into behaviour (Sheeran & Webb, 2016). Whether FCC affects prosocial behaviour similarly should be addressed in future studies.

Future research should also address the limitations of our work. First, further investigation is needed to generalize FCC's structure across different cultural and social settings. Second, while we focused on the similarity component of FCC, future studies could explore manipulations of vividness and positivity. Third, small effects of FCC on intergroup relations may accumulate over time, but empirical validation is required, ideally through longitudinal methods, to assess how these effects fluctuate (Funder & Ozer, 2019). Fourth, another interesting area is FCC's role in social identity formation, which may foster inclusivity and emphasize valued ingroup traits (Brewer & Pierce, 2005; Gaertner & Dovidio, 2000). Relatedly, we argued that FCC and PCC affect intergroup relations differently. Future work might focus on disentangling this relationship and understanding how both variables affect social self‐esteem, national identity, and intergroup relations. Fifth, FCC can apply to other groups beyond national identity, such as religious or family affiliations (Herrera et al., 2011; Sani, 2005; Warner et al., 2016). Therefore, FCC should be studied in varied group settings. Finally, our study centred on anxiety‐related emotions, but examining positive emotions like pride (Sullivan, 2014) and hope (Cohen‐Chen et al., 2014) could reveal additional benefits for FCC in enhancing intergroup dynamics.

To conclude, the present work provided evidence that perceiving the ingroup as an entity that endures in the future might be beneficial to increase intergroup cooperation in the present. Expecting that the ingroup traditions, values, and norms will stay the same in future times could produce fewer concerns that external agents might damage the ingroup and, thus, greater openness to different social entities in the present. Like Claudius two millennia ago, we could use the belief that our ways will not change to make us more welcoming to others different from us.

## AUTHOR CONTRIBUTIONS

**Andrej Simić:** Conceptualization; investigation; writing – original draft; writing – review and editing; methodology; data curation; formal analysis. **Simona Sacchi:** Conceptualization; investigation; writing – review and editing; methodology; supervision. **Marco Perugini:** Supervision; conceptualization; investigation; methodology; writing – review and editing.

## CONFLICT OF INTEREST STATEMENT

There are no conflicts of interest to declare.

## Supporting information


Appendix S1

## Data Availability

The data that support the findings of this study are openly available in OSF at https://osf.io/ezy5p/?view_only=efaff11e2fc347a5877d6d07edb7773a.

## Footnotes

^1^
In Study 2, we originally aimed to provide evidence of a positive effect of FCC on outgroup prosocial expectations. However, our manipulation was not effective. Here, we present data on a correlational level, while we include the full description of the experimental procedure and subsequent data analysis in Appendix S1.

^2^
The videos presented to participants in both conditions are publicly available on our OSF directory.

^3^
Following our preregistration, we also fitted a simple mediation model (Model 4, Hayes, 2018) that pointed to a similar conclusion. We present the estimated model in Appendix S1.

^4^
This part of the analysis diverged from our preregistration. Originally, when running the preregistered three simple moderation models (Model 1; Hayes, 2018) our analysis points to a similar conclusion: the manipulation was effective for participants with higher levels of national attachment, but not for low identifiers. We report on these models in Appendix S1.

^5^
However, the interpretation of the indirect effect (namely the ab and abd paths) should remain on the correlational level as Hauser et al. (2018) note.
