# Supplementary material for: Our ways will not change: Future collective continuity increases present prosocial considerations
Source: Br J Soc Psychol. 2024 Dec 31;64(1):e12847. doi: 10.1111/bjso.12847 (PMC11687412; doi:10.1111/bjso.12847)

**Supplemental Materials for:**

**Our Ways Will Not Change: National Collective Future Continuity Increases Present Prosocial Considerations**

**Contents**

Study 1 Additional Analyses, pages 4-7

Study 2 Additional Analyses, pages 7-11

Study 3 Additional Analyses, pages 11-12

**Tables**

*Table 1.* Study 1: Component loadings of the proposed four-component structure of group-level MFQ, pages 13-14

*Table 2.* Study 1: Component loadings of the proposed two-component structure of group-level MFQ, pages 15-16

*Table 3.* Study 1: Means, standard deviations and correlations of the study variables, page 17

*Table 4.* Study 2: Means and standard deviations of the study variables as a function of the temporal and ingroup-frames, page 18

*Table 5.* Summary of univariate tests of manipulation effects on manipulation check and dependent variables, page 19

*Table 6.* Study 3: model fit indices and estimated regression coefficients of Condition, national identity, and Condition x national identity interactions as predictors of outgroup prejudice (Model 1), outgroup emotions (Model 2), and collective actions (Model 3) as outcome variables, page 20

*Table 7.* Study 3: estimated effects of Condition on outgroup prejudice (Model 1), outgroup emotions (Model 2), and collective actions (Model 3) as a function of different levels of national identity, page 21

*Figure 1*. Study 1: mediation model containing CFC components as predictors, outgroup prosocial beliefs as the outcome variable, and prosocial collective efficacy as a mediator, page 22

*Figure 2*. Study 2: mediation model containing CFC components are predictors, outgroup prejudice and ingroup prosocial behavior as outcome variables, and collective angst as a mediator, page 23

*Figure 3*. Study 3: mediation model containing Condition as the predictor, collective angst as the mediator, and outgroup prejudice as the outcome variable, page 24

*Figure 4*. Study 3: Simple slope analysis depicting the effect of Condition on outgroup prejudice (Model 1), outgroup emotions (Model 2), and collective action intentions (Model 3) as a function of different levels of national identity, page 25

**Study 1 Additional Analyses**

In Study 1, we were also interested in the relationship between future self-continuity and collective future continuity facets with moral foundations on the individual and group level. Here we report the results of these additional analyses and the description of measures not included in the main body of the manuscript.

*Future Self-Continuity Questionnaire (FSCQ)*. Sokol and Serper (2020) proposed an alternative measure of future self-continuity. The 10-item questionnaire consists of 3 subscales: Similarity to the future self (4 items, e.g., How similar are your beliefs now to what they will be like 10 years from now?), Vividness of the future self (3 items, e.g., How vividly can you imagine what you will be like in 10 years from now?), and Positive affect to the future self (3 items, e.g., Do you like what you will be like 10 years from now?). Participants are asked to indicate their level of agreement/disagreement with the items on a seven-point Likert scale (1 = completely different/not at all, 7 = exactly the same/perfectly).

*Prosocial Collective Efficacy Scale* (PCES; Cuadrado et al., 2016). We measured the confidence that the ingroup (Italians) can behave cooperatively using the PCES developed by Cuadrado and colleagues (2016). The scale consisted of five items where participants are asked to determine the level at which their ingroup is capable of prosocial behavior (e.g., Italians can share resources). The responses were given on a 7-pointscale (1 = strongly disagree, 7 = strongly agree).

*Moral Foundations – Individual Level*. We assessed individual levels of moral foundations using the Italian versions of the MFQ (Bobbio et al., 2011). The measure is composed of two 15-item subscales. The Relevance subscale contains behaviors that are assessed by how important they are when judging whether something is moral (e.g., Whether or not someone suffered emotionally). In the Judgement subscale (e.g., It can never be right to kill a human being.) Participants are asked to provide their levels of agreement/disagreement with the items. All items were answered by using a 7-point Likert scale (1 = not relevant at all /strongly disagree, 7 = extremely relevant/strongly agree). By following Graham and colleagues (2011), we have combined individual items that measure Harm (6 items) and Fairness (6 items) into a composite individualizing MFs index. Likewise, we created a composite score of binding MFs by combining the items related to Loyalty (6 items), Authority (6 items), and Purity (6 items).

*Moral Foundations – Group Level.* Winget and Tindale (2020) adapted the individual-level MFQ to the group setting. Their measure contains the same subscales (Judgement and Relevance), where the items are modified to highlight group membership. The participants are asked to determine how much each item is relevant when making moral judgments about *ingroup members* (Relevance subscale; Whether or not *they* suffered emotionally) and to indicate their level of agreement/disagreement with the items about *their ingroup members* (Judgement subscale; I believe *they* are proud of their country's history) on a 7-point Likert scale (1 = strongly agree, 7 = strongly disagree). Using the same method as the individual-level MFQ, we formed group-level individualizing and binding moral foundations indices.

**Results**

Data analysis was done inside the R statistical software (R Core Team, 2020). We computed the correlations between variables using the psych package. (Revelle, 2019). The principal component analysis was done using the psych (Revelle, 2019) and GPArotation (Bernaards, Coen & Jennrich, 2005) packages.

Here, we report all additional analyses not present in the manuscript. Since we have adopted this measure for the first time in an Italian context, we have examined the component structure of the group level-MFQ. The scree plot and parallel analysis (Horn, 1965) recommended four components for retention. The four components explained approximately 53% of participants' responses variance. The component loadings are shown in Table 1. The first six items from the Relevance subscale loaded highly on Component 3, resembling the individualizing moral foundations related to the Relevance subscale. The binding moral foundations part of the Relevance subscale loaded highly on Component 2 and two items from the Judgement subscale. Seven items from the Judgement subscale loaded highly on Component 1, while three items in the same subscale loaded on Component 4. The remaining three items loaded highly on more than one component.

Because a four-component solution was difficult to interpret, we ran a more theoretically sound two-component solution. The two components explained approximately 40% of data variance. The component loadings for a two-component solution are presented in Table 2. Nine items from the Relevance subscale loaded highly on Component 2. Component 1 was loaded by four times from the Relevance subscale and nine from the Judgement subscale. The remaining items loaded highly on both components. In other words, the components did not correspond to the expected Individualizing and Binding moral foundations subscales. Because of the difficulty of interpreting this measure's component structure, we decided against including it in our main analyses for Studies 1 and 2.

Table 3 contains the correlations not reported in the main manuscript. Collective future similarity correlated positively with Future self-continuity similarity and vividness facets. The Vividness component was positively related to all three future self-continuity facets, while Positivity with the future collective correlated positively only with Future self-vividness. Furthermore, all three collective future continuity components were positively related to both Individualizing and Binding moral foundations on the group level. We found a positive correlation only with Binding moral foundations and the Vividness component on the individual level. All other correlations between the collective future similarity facets and other variables did not reach significance.

**Mediation Effect of Prosocial Collective Efficacy**

We initially planned to also study the how prosocial collective efficacy beliefs mediate the relationship between the CFC components and outgroup prosocial beliefs. However, our examination of the bivariate relations between the study variables showed that the correlation between prosocial collective efficacy and outgroup prosocial beliefs was unusually high (*r* = .83, see Table 3). Therefore, the two variables may not be measuring empirically distinct constructs. When the mediator and outcome variable might measure very similar constructs, we cannot infer a mediation effect even from a significant mediation model because the independent variable may simply affect two highly correlated psychological processes (see Spencer et al., 2005). Therefore, we decided against examining our hypothesis with a mediation model and report it here in an exploratory fashion.

Figure 1 shows the tested mediational model. We have included the three CFC components as predictors, while collective prosocial efficacy and outgroup prosocial beliefs served as the mediator and outcome variables, respectively. The bootstrapping procedure (Hayes, 2018) on 10,000 bootstrapped samples was used to test the significance of the indirect effects. The total and indirect effects through collective prosocial efficacy beliefs of the similarity (total: *b* = .27, 95% CI [.05, .52] indirect: *b* = .29, 95% CI [.13, .48]) and positivity (total: *b* = .47, 95% CI [.23, .66]; indirect: *b* = .28, 95% CI [.06, .49]) components were significant. On the other hand, the same effects when considering vividness as the predictor variable were not significant, total: *b* = -.14, 95% CI [-.39, .11]; indirect: *b* = -.02, 95% CI [-.21, .18].

**Additional Analyses Study 2**

We originally developed Study 2 to investigate the effect of an experimental manipulation to increase collective future continuity on a) ingroup prosocial behavior and b) prejudice towards the outgroup. We will briefly describe the experimental procedure and the subsequent multi and univariate analyses.

**Method**

**Materials and Procedure**

After giving informed consent, participants we randomly assigned to four experimental groups. To manipulate CFC, we adapted the manipulation devised by Hershfield and colleagues (2012) to increase FSC to the framework of CFC. The first group of participants focused on the similarities between Italians 50 years in the future and now (group future-focused similarity condition), and the second experimental group thought about Italians in the present (a group present-focused condition). The third group focused on the similarities between public transport 50 years in the future and now (a neutral future-focused similarity condition), and the fourth one thought about the present characteristics of public transport (a neutral present-focused condition). Participants in all four groups received the following instructions:

"Some recent studies in the field of social sciences [in the field of technology] have identified a series of characteristics that define a specific culture (e.g., beliefs, language, traditions, and rites, food, diet, religion) [measures of transportation (e.g., comfort, speed, environmental impact]. We ask you to think how the Italian culture and society [measures of transport] will be in 50 years [is now] and list in the box below all aspects of Italians [measures of transport] that will be similar to now [of the present moment]. Specifically, think about the characteristics of the Italian national group [measures of transport] that will remain stable in the future [describe them]. Take two minutes to think about and write the answers to this question. You will be able to advance to the next page after the timer (shown in the upper left corner) expires. You can take more than two minutes if you need the additional time". After finishing their respective tasks, participants completed the remaining measures used in this study: Collective Future Continuity Questionnaire (CFCQ), Prosocial Behavior Scale for Adults ([PBSA]; Carprara et al., 2006), the adapted Modern Prejudice Scale ([MDS]; McConahay, 1986), and the Collective Angst Scale ([CSA]; Wohl & Branscombe, 2009). Here, we describe the PSA since we did not include this measure in our main manuscript.

*Prosocial Behavior Scale for Adults ([PBSA]; Caprara et al. 2005)*. The PBSA measures individual differences in general prosocial behavior. In this 16-item measure, participants are presented with various present-day situations that require helping others (e.g., being available for volunteer activities to help those in need). We modified the initial instructions to measure participants' expectations of the in-groups' prosocial behavior (''To what extent Italians *should* engage in following behaviors). Participants responded how much each behavior described in the item applies to them on a 7-point scale (1 = never/almost never true, 7 = always/almost always true).

**Results**

**Manipulation Check**

Table 4 contains the relevant descriptive statistics for our study variables by temporal (present vs. future) and ingroup (ingroup vs. neutral) focus conditions. We conducted a 2 (present vs. future) X 2 (ingroup vs. netural) MANOVA with the three collective future facets as dependent variables. We found no main effects of ingroup [*F* (3, 194) = .41, *p* = .742, *η^2^* = .01] or temporal frame [*F* (3, 194) = .13, *p* = .944, *η^2^* < .01] as well as interaction [*F* (3, 194) = .63, *p* = .598, *η^2^* = .01]. Regardless, we decided to explore our manipulation’s effects further by employing three separate univariate follow-ups which are shown in Table 5. Similarly, we found no significant main or interactions effects when considering the three collective future continuity subscales as separate dependent variables (all *p*s > .344). In other words, our manipulation did not successfully increase participants’ collective future continuity.

**Ingroup Prosocial Behavior and Outgroup Prejudice**

We conducted two separate 2 (present vs. future) X 2 (ingroup vs. neutral) ANOVAs with ingroup prosocial behavior and outgroup prejudice as dependent variables. As with the manipulation check, we observed no significant main and interaction effects (all *p*s > .114; see Table 5).

**Including General Prosocial Expectations into Study 2 Mediation Model**

In an exploratory fashion, we attempted to understand whether the effect of CFC is specific to outgroup beliefs or that it can be generalized to more general expectations of ingroup prosocial behavior. Therefore, we included the PBSA as an additional outcome variable in the model we described in the main manuscript.

The proposed mediation model is represented in Figure 2. CFC components were included as predictors, collective angst as the mediator, prejudice towards the outgroup, and prosocial intention towards the ingroup as outcome variables. We tested the indirect effects by following the bootstrapping procedure (Hayes, 2018) on 10,000 bootstrapped samples. For future collective similarity, collective angst significantly mediated the effect on prejudice towards the outgroup (total: *b* = -.08, 95% CI [-.18, .02]; indirect: *b* = -.06, 95% CI [-.11, -.01]), but not on prosocial expectations towards the ingroup (total: *b* = -.01, 95% CI [-.13, .12]; indirect: *b* = .02, 95% CI [-.01, .05]). The indirect effect of collective angst was not significant when considering the relationship between future collective vividness and both outgroup prejudice (total: *b* = -.14, 95% CI [-.28, -.01], indirect: *b* = -.05, 95% CI [-.12, .02]) and ingroup prosocial expectations (total: *b* = -.06, 95% CI [-.21, .07], indirect: *b* = .01, 95% CI [-.01, .05]). Finally, collective angst significantly mediated the effect of positivity of the future collective on outgroup prejudice (total: *b* = -.18, 95% CI [-.31, -.07], indirect: *b* = -.07, 95% CI [-.14, -.02]), but not on expectations to behave prosocially towards the ingroup (total: *b* = .09, 95% CI [-.02, .21]; indirect: *b* = .02, 95% CI [-.01, .07]).

We have compared mediation effects of collective angst separately for all three CFC components. The indirect effect on outgroup prejudice was stronger than the same effect on ingroup prosocial expectations for the similarity (*b* = .08, 95% CI [.02, .16]) and positivity (*b* = .10, 95% CI [.02, .20] components. However, we found no significant differences between the indirect effects when considering vividness as the predictor variable, *b* = .06, 95% CI [-.02, .16].

**Additional Analyses Study 3**

**Simple Mediation Model**

As stated in the main manuscript, we also fitted a simple mediation model (Model 4, Hayes 2018) that included Condition as the predictor, collective angst as the mediator, and modern prejudice as the outcome variable. The estimated model is presented in Figure 1. We used the bootstrapping method to determine each path’s significance (10,000 bootstrapped samples). The estimated mediation effect of collective angst did not include zero signifying statistical significance, *b* = -.25, 95% CI [-.44, -.08]. Therefore, our analysis suggests that lower levels of collective angst might explain our manipulation effect.

**Moderation Models**

***The Moderating Effect of National Identity***

Following our preregistration, we conducted three moderated regression models containing manipulation as the independent, national attachment as moderator, and outgroup prejudice (Model 1), outgroup anxiety (Model 2), collective actions intentions (Model 3) as dependent variables. Prior to conducting the analysis we mean-centered participants scores on the NIS (Baron & Kenny, 1986). Table 6 contains the estimated regression coefficients four our three moderated regression models. All moderation models were significant explaining more than 17% of dependent variable’s variance. Furthermore, we identified significant Condition x national identity interactions in all three models. We investigated these interactions further with simple slope analysis (see Figure 4). As Table 7 shows, we found a significant effect of condition higher, but not on lower levels of national identity when considering all three of our dependent variables. In line with our moderation hypothesis, our experimental manipulation seemed to have a detrimental effect on proxies of negative outgroup in the case of high identifiers.

***The Moderating Effect of Political Identity***

Additionally, we explored the moderating effect of political identity in an exploratory fashion. As in the previous case, we developed three moderated regression models containing manipulation as the independent, political identity as moderator, and outgroup prejudice (Model 1), outgroup emotions (Model 2), collective actions intentions (Model 3) as dependent variables. Prior to conducting the analysis we mean-centered participants reported political identity (Baron & Kenny, 1986). Table 8 contains the estimated regression coefficients four our three moderated regression models. All moderation models were significant explaining more than 33% of dependent variable’s variance. Furthermore, we identified significant Condition x political identity interactions in all three models. We investigated these interactions further with simple slope analysis (see Figure 5). As Table 9 shows, we found on higher level of political identity (more right-wing oriented), condition decreased prejudice towards immigrants. Unexpectedly, on higher levels of political identity (more left-wing oriented), condition increased prejudice. For the other two models, condition decreased outgroup anxiety and increased outgroup collective action on lower levels of political identity. On lower levels, the effect of condition was not significant.

**Table 1.**

*Study 1: Component loadings of the proposed four-component structure of group-level MFQ.*

| *Relevance subscale* | C1 | C2 | C3 | C4 |
| --- | --- | --- | --- | --- |
| Whether or not they suffered emotionally | 0.13 | -0.05 | 0.77 | -0.03 |
| Whether or not they cared for someone weak or vulnerable | 0.00 | 0.05 | 0.78 | 0.02 |
| Whether or not they was cruel | -0.09 | 0.11 | 0.78 | 0.17 |
| Whether or not they was denied his or her rights | -0.09 | 0.07 | 0.63 | -0.17 |
| Whether or not they people were treated differently from others | 0.06 | -0.09 | 0.78 | 0.03 |
| Whether or not they was denied his or her rights | -0.09 | 0.03 | 0.74 | -0.12 |
| Whether or not they showed a lack of loyalty | -0.14 | 0.75 | 0.22 | -0.13 |
| Whether or not they did something to betray his or her group | 0.10 | 0.67 | 0.08 | -0.08 |
| Whether or not they action showed love for his or her country | 0.07 | 0.73 | -0.19 | 0.11 |
| Whether or not they conformed to the traditions of society | 0.41 | 0.43 | -0.16 | 0.29 |
| Whether or not they showed a lack of respect for authority | 0.02 | 0.68 | 0.01 | 0.12 |
| Whether or not their action caused chaos or disorder | 0.04 | 0.33 | 0.40 | 0.16 |
| Whether or not they did something disgusting | -0.18 | 0.43 | 0.49 | 0.05 |
| Whether or not they violated standards of purity and decency | 0.32 | 0.59 | -0.01 | 0.04 |
| Whether or not they acted in a way that God would approve of | 0.44 | 0.33 | -0.13 | 0.11 |
| *Judgment subscale* |  |  |  |  |
| One of the worst things Italians could do is hurt a defenseless animal. | 0.24 | 0.41 | -0.01 | -0.23 |
| It can never be right for Italians to kill a human being. | 0.69 | 0.05 | 0.07 | -0.31 |
| Compassion for those who are suffering is the most crucial virtue of Italians. | 0.70 | 0.15 | -0.02 | -0.09 |
| Justice is the most important requirement for Italians. | 0.72 | 0.20 | -0.02 | -0.03 |
| When the government makes laws, the number one principle should be ensuring that every Italian is treated fairly. | 0.42 | 0.20 | 0.21 | -0.41 |
| I believe that Italians would think it's morally wrong that rich children inherit a lot of money while poor children inherit nothing. | 0.63 | -0.13 | -0.08 | 0.16 |
| It is more important that they Italians are team player than to express oneself. | 0.88 | -0.09 | 0.00 | 0.05 |
| I think Italians are proud of my country's history. | 0.32 | 0.05 | 0.11 | 0.27 |
| Italians should be loyal to their family members, even when they have done something wrong. | 0.59 | 0.11 | -0.14 | 0.11 |
| Italian men and women each have different roles to play in society. | -0.16 | 0.23 | -0.18 | 0.68 |
| If any Italian were a soldier and disagreed with my commanding officer's orders, I would obey anyway because that is my duty. | 0.48 | 0.00 | 0.13 | 0.43 |
| Respect for authority is something all Italian children need to learn. | 0.09 | 0.74 | 0.00 | 0.04 |
| Italians should not do things that are disgusting, even if no one is harmed. | -0.10 | 0.58 | 0.21 | -0.10 |
| I would call some acts Italians do wrong on the grounds that they are unnatural. | 0.10 | -0.14 | 0.39 | 0.64 |
| Chastity is an important and valuable virtue for Italians. | 0.47 | 0.01 | -0.09 | 0.54 |

**Table 2.**

*Study 1: Component loadings of the proposed two-component structure of group-level MFQ.*

| *Relevance subscale* | C1 | C2 |
| --- | --- | --- |
| Whether or not they suffered emotionally | -0.04 | 0.66 |
| Whether or not they cared for someone weak or vulnerable | -0.07 | 0.73 |
| Whether or not they was cruel | -0.06 | 0.75 |
| Whether or not they was denied his or her rights | -0.18 | 0.67 |
| Whether or not they people were treated differently from others | -0.11 | 0.65 |
| Whether or not they was denied his or her rights | -0.2 | 0.73 |
| Whether or not they showed a lack of loyalty | 0.31 | 0.64 |
| Whether or not they did something to betray his or her group | 0.49 | 0.42 |
| Whether or not they action showed love for his or her country | 0.61 | 0.17 |
| Whether or not they conformed to the traditions of society | 0.76 | -0.06 |
| Whether or not they showed a lack of respect for authority | 0.51 | 0.33 |
| Whether or not their action caused chaos or disorder | 0.26 | 0.49 |
| Whether or not they did something disgusting | 0.08 | 0.69 |
| Whether or not they violated standards of purity and decency | 0.69 | 0.24 |
| Whether or not they acted in a way that God would approve of | 0.66 | -0.05 |
| *Judgment subscale* |  |  |
| One of the worst things Italians could do is hurt a defenseless animal. | 0.41 | 0.21 |
| It can never be right for Italians to kill a human being. | 0.52 | 0.03 |
| Compassion for those who are suffering is the most crucial virtue of Italians. | 0.69 | -0.04 |
| Justice is the most important requirement for Italians. | 0.76 | -0.03 |
| When the government makes laws, the number one principle should be ensuring that every Italian is treated fairly. | 0.33 | 0.31 |
| I believe that Italians would think it's morally wrong that rich children inherit a lot of money while poor children inherit nothing. | 0.53 | -0.29 |
| It is more important that they Italians are team player than to express oneself. | 0.72 | -0.22 |
| I think Italians are proud of my country's history. | 0.39 | 0.01 |
| Italians should be loyal to their family members, even when they have done something wrong. | 0.65 | -0.2 |
| Italian men and women each have different roles to play in society. | 0.27 | -0.17 |
| If any Italian were a soldier and disagreed with my commanding officer's orders, I would obey anyway because that is my duty. | 0.55 | -0.05 |
| Respect for authority is something all Italian children need to learn. | 0.59 | 0.36 |
| Italians should not do things that are disgusting, even if no one is harmed. | 0.24 | 0.53 |
| I would call some acts Italians do wrong on the grounds that they are unnatural. | 0.16 | 0.13 |
| Chastity is an important and valuable virtue for Italians. | 0.62 | -0.28 |

**Table 3.**

*Study 1: Means, standard deviations, and correlations of the study variables.*

| Variable | *M* | *SD* | 1 | 2 | 3 | 4 | 5 | 6 | 7 | 8 | 9 | 10 |
| --- | --- | --- | --- | --- | --- | --- | --- | --- | --- | --- | --- | --- |
| Collective future similarity (1) | 3.89 | 1.17 | .94 |  |  |  |  |  |  |  |  |  |
| Collective future vividness (2) | 3.66 | 1.01 | .57** | .92 |  |  |  |  |  |  |  |  |
| Collective future Positivity (3) | 3.9 | .99 | .35** | .49** | .92 |  |  |  |  |  |  |  |
| Future self-similarity (4) | 4.75 | .77 | .20** | .22** | -.01 |  |  |  |  |  |  |  |
| Future self-vividness (5) | 4.34 | .94 | .26** | .46** | .27** | .54** | .80 |  |  |  |  |  |
| Future self-positivity (6) | 4.59 | .91 | -.01 | .20** | .07 | .54** | .57** | .89 |  |  |  |  |
| Individual - level Individualizing moral foundations (7) | 5.46 | .58 | -.09 | -.05 | -.01 | .18** | -.02 | .09 | .75 |  |  |  |
| Individual - level Binding moral foundations (8) | 4.54 | .68 | -.09 | .16* | .09 | .22** | .14* | -.07 | .33** | 0.82 |  |  |
| Group - level Individualizing moral foundations (9) | 5.25 | .58 | .20** | .26** | .26** | .00 | -.03 | -.06 | .50** | .45** | 0.71 |  |
| Group - level Binding moral foundations (10) | 4.59 | .73 | .48** | .28** | .27** | .14* | .16* | -.05 | .12 | .78** | .57 | 0.82 |

*Note*. M and SD represent the means and standard deviations. * indicates *p* < .05. ** indicates *p* < .01. Values in the brackets indicate alpha coefficients for each scale.

**Table 4.**

*Study 2: Means and standard deviations of the study variables as a function of the temporal and ingroup-frames.*

|  |  | Similarity | | Vividness | | Positivity | | Ingroup  Prosocial  Behavior | | Outgroup  Prejudice | | Collective  Angst | |
| --- | --- | --- | --- | --- | --- | --- | --- | --- | --- | --- | --- | --- | --- |
| Temporal frame | Ingroup-frame | *M* | *SD* | *M* | *SD* | *M* | *SD* | *M* | *SD* | *M* | *SD* | *M* | *SD* |
| Future | Ingroup | 3.85 | 1.37 | 3.57 | 1.09 | 3.64 | 1.60 | 5.63 | .72 | 2.18 | .88 | 2.25 | 1.56 |
|  | Neutral | 3.56 | 1.16 | 3.53 | 1.13 | 3.77 | 1.15 | 5.37 | .95 | 2.12 | .98 | 2.11 | 1.06 |
| Present | Ingroup | 3.75 | 1.28 | 3.47 | .97 | 3.86 | 1.23 | 5.31 | .95 | 2.23 | .97 | 2.00 | 1.01 |
|  | Neutral | 3.69 | 1.31 | 3.54 | 1.14 | 3.70 | 1.25 | 5.44 | .81 | 1.96 | .90 | 2.15 | 1.31 |

*Note*. M and SD represent the means and standard deviations.

**Table 5.**

*Summary of univariate tests of manipulation effects on manipulation check and dependent variables.*

|  | Variable | | | | | | | | | | | | | | |
| --- | --- | --- | --- | --- | --- | --- | --- | --- | --- | --- | --- | --- | --- | --- | --- |
|  | Similarity | | | Vividness | | | Positivity | | | Ingroup  Prosocial  Behavior | | | Outgroup  Prejudice | | |
| Effect | *F* | *p* | *η^2^* | *F* | *p* | *η^2^* | *F* | *p* | *η^2^* | *F* | *p* | *η^2^* | *F* | *p* | *η^2^* |
| Temporal Frame | .90 | .344 | .00 | .01 | .914 | .00 | .01 | .910 | .00 | .18 | .670 | .00 | 1.60 | .208 | .01 |
| Ingroup Frame | .02 | .895 | .00 | .06 | .803 | .00 | .12 | .732 | .00 | .891 | .346 | .00 | .27 | .606 | .00 |
| Temporal Frame x Ingroup Frame | .42 | .518 | .00 | .13 | .716 | .00 | .57 | .453 | .00 | 2.53 | .114 | .01 | .64 | .426 | .00 |

**Table 6.**

*Study 3: model fit indices and estimated regression coefficients of Condition, national identity, and Condition x national identity interactions as predictors of outgroup prejudice (Model 1), outgroup emotions (Model 2), and collective actions (Model 3) as outcome variables.*

| Indirect effect | Outgroup prejudice (Model 1) | | | Outgroup emotions (Model 2) | | Collective actions (Model 3) | |
| --- | --- | --- | --- | --- | --- | --- | --- |
| *R^2^* |  | .24 |  | .25 | | .17 | |
| *R_adj2_* |  | .23 |  | .24 | | .16 | |
| *F* (3, 248) |  | 25.78*** |  | 27.11*** | | 17.06*** | |
|  | *b* | *t* | | *b* | *t* | *b* | *t* |
| Intercept | 2.57 | 28.49** | | 3.56 | 35.75*** | 3.33 | 24.29*** |
| Condition (Continuity vs. Discontinuity) | -.30 | -2.36* | | -.64 | -4.52*** | .42 | 2.16* |
| National identity | .56 | 8.08*** | | .50 | 7.83*** | -.65 | -6.08* |
| Condition x National Identity | -.37 | -3.83** | | -.47 | -4.41*** | .31 | 2.10* |

*Note. R^2^* = value of the coefficient of determination. *R_adj_^2^* = value of the adjust coefficient of determination. *F* = value of the F statistic (degrees of freedom are shown in brackets). *b* = value of the unstandardized regression coefficient. * indicates *p* < .05. ** indicates *p* < .01. *** indicates *p* < .001.

**Table 7.**

*Study 3: estimated effects of Condition on outgroup prejudice (Model 1), outgroup emotions (Model 2), and collective actions (Model 3) as a function of different levels of national identity.*

|  | Model 1  (outgroup prejudice) | | Model 2  (outgroup emotions) | | Model 3  (collective actions) | |
| --- | --- | --- | --- | --- | --- | --- |
| Levels of national identity | *b* | t | *b* | t | *b* | t |
| Lower (-1 SD) | .19 | 1.06 | -.01 | -.07 | .01 | .03 |
| Higher (+1 SD) | -.79 | -4.37*** | -1.26 | -6.30*** | .83 | 3.00** |

*Note.* *b* = value of the unstandardized regression coefficient. * indicates *p* < .05. ** indicates *p* < .01. *** indicates *p* < .001.

**Table 8.**

*Study 3: model fit indices and estimated regression coefficients of Condition, political identity, and Condition x political identity interactions as predictors of outgroup prejudice (Model 1), outgroup emotions (Model 2), and collective actions (Model 3) as outcome variables.*

| Indirect effect | Outgroup prejudice (Model 1) | | | Outgroup emotions (Model 2) | | Collective actions (Model 3) | |
| --- | --- | --- | --- | --- | --- | --- | --- |
| *R^2^* |  | .44 |  | .34 | | .39 | |
| *R_adj2_* |  | .44 |  | .33 | | .39 | |
| *F* (3, 247) |  | 65.60*** |  | 42.40*** | | 53.36*** | |
|  | *b* | *t* | | *b* | *t* | *b* | *t* |
| Intercept | 2.65 | 34.15** | | 3.64 | 38.61*** | 3.20 | 27.16*** |
| Condition (Continuity vs. Discontinuity) | -.30 | -2.71* | | -.66 | -4.92*** | .41 | 2.42* |
| Political identity | .64 | 12.46*** | | .60 | 9.83*** | -.82 | -10.58*** |
| Condition x National Identity | -.35 | -5.05** | | -.41 | -4.93 | .35 | 3.31** |

*Note. R^2^* = value of the coefficient of determination. *R_adj_^2^* = value of the adjust coefficient of determination. *F* = value of the F statistic (degrees of freedom are shown in brackets). *b* = value of the unstandardized regression coefficient. * indicates *p* < .05. ** indicates *p* < .01. *** indicates *p* < .001. One participant was deleted due to a missing value on the political identity variable.

**Table 9.**

*Study 3: estimated effects of Condition on outgroup prejudice (Model 1), outgroup emotions (Model 2), and collective actions (Model 3) as a function of different levels of political identity.*

|  | Model 1  (outgroup prejudice) | | Model 2  (outgroup emotions) | | Model 3  (collective actions) | |
| --- | --- | --- | --- | --- | --- | --- |
| Levels of national political identity | *b* | t | *b* | t | *b* | t |
| Lower (-1 SD) | .34 | 2.18* | .09 | .49 | -.22 | -.96 |
| Higher (+1 SD) | -.77 | -4.99*** | -1.22 | -6.50*** | .89 | 3.74** |

*Note.* *b* = value of the unstandardized regression coefficient. * indicates *p* < .05. ** indicates *p* < .01. *** indicates *p* < .001.

**Figure 1.**

*Study 1: mediation model containing CFC components as predictors, outgroup prosocial beliefs as the outcome variable, and prosocial collective efficacy as a mediator..*

*
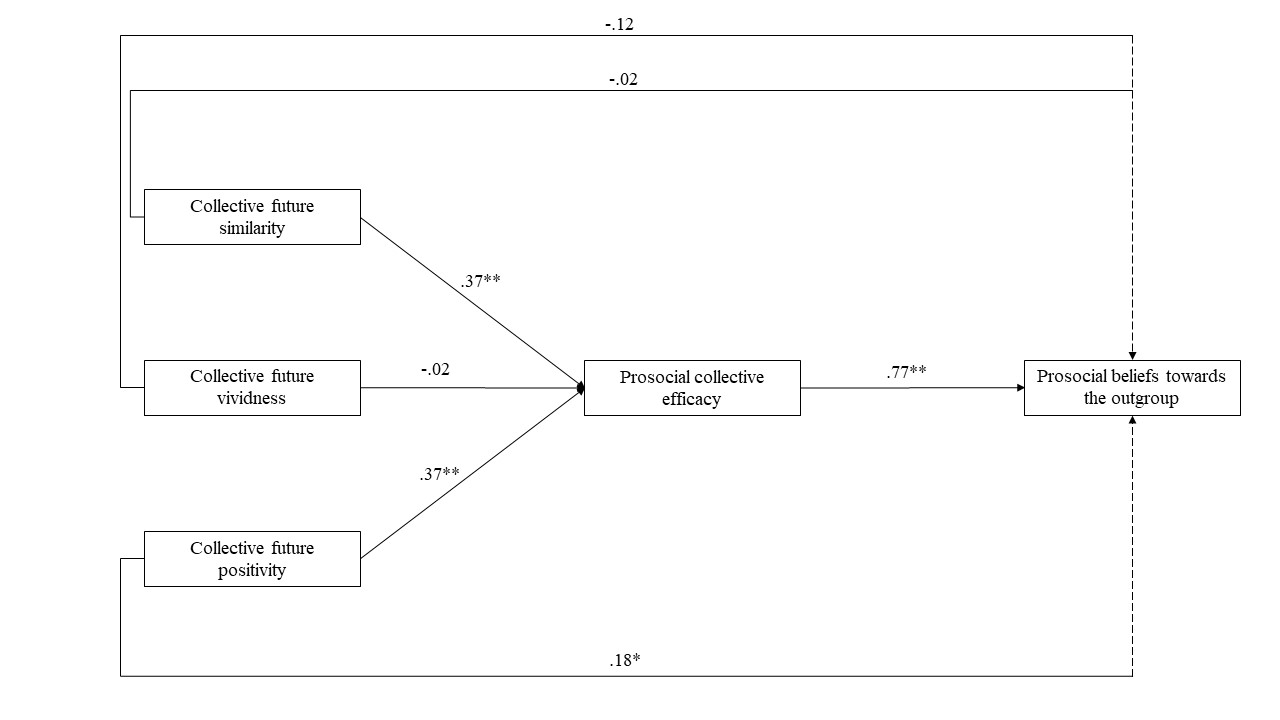
*

Note. The figure contains unstandardized regression weights as representations of path coefficients. * indicates *p < .01*. ** indicates *p* < .001

**Figure 2.**

*Study 2: mediation model containing CFC components are predictors, outgroup prejudice and ingroup prosocial behavior as outcome variables, and collective angst as a mediator.*

*
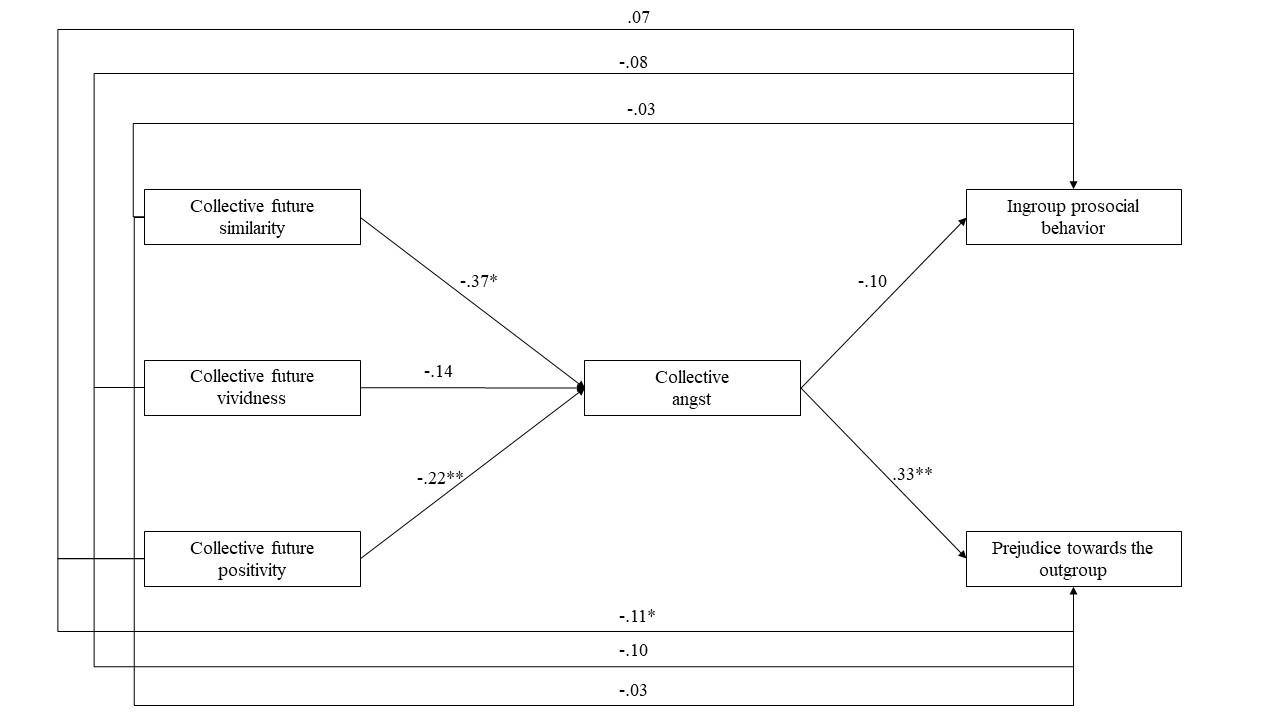
*

Note. The figure contains unstandardized regression weights as representations of path coefficients. * indicates *p < .01*. ** indicates *p* < .001

**Figure 3.**

*Study 3: mediation model containing Condition as the predictor, collective angst as the mediator, and outgroup prejudice as the outcome variable.*


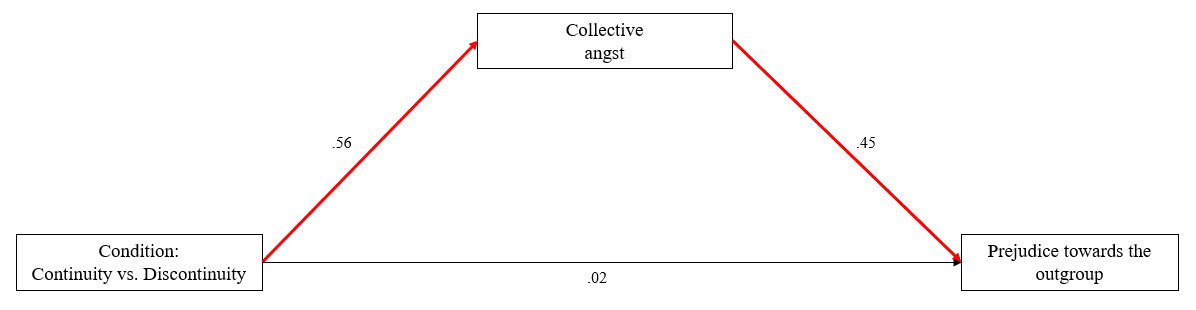


Note. The figure contains unstandardized regression weights as representations of path coefficients. Red lines represent significant effects.

**Figure 4.**

*Study 3: Simple slope analysis depicting the effect of Condition on outgroup prejudice (Model 1), outgroup emotions (Model 2), and collective action intentions (Model 3) as a function of different levels of national identity.*


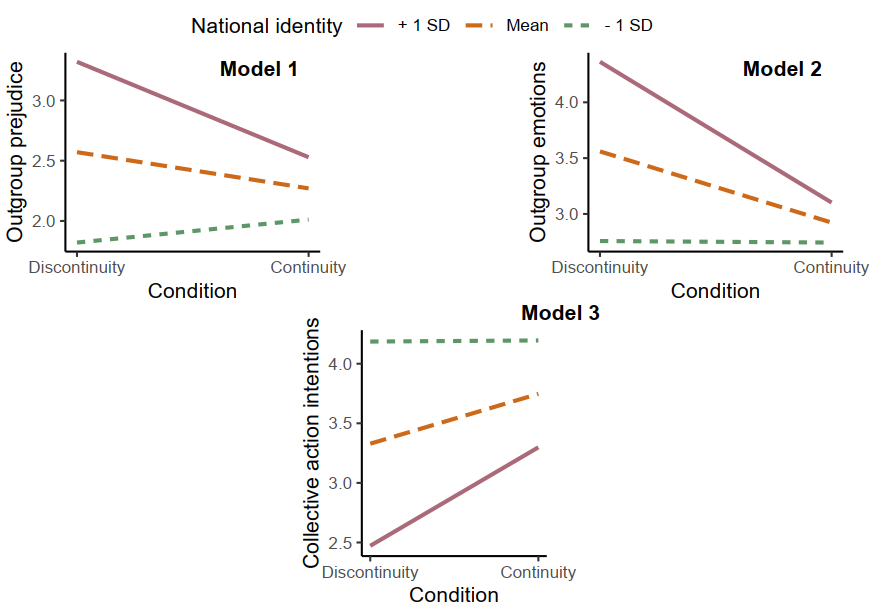


**Figure 5.**

*Study 3: Simple slope analysis depicting the effect of Condition on outgroup prejudice (Model 1), outgroup emotions (Model 2), and collective action intentions (Model 3) as a function of different levels of political identity identity.*


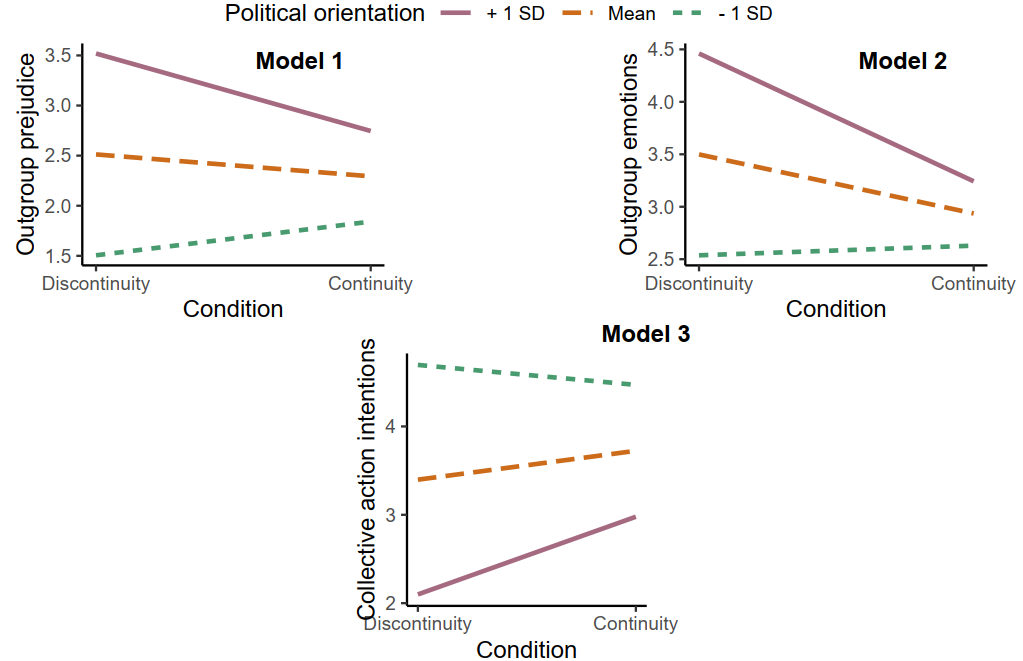

Supplement: Supplementary file 1 — Appendix S1 [file BJSO-64-0-s001.docx]
